# Multiomics Analysis of Exportin Family Reveals XPO1 as a Novel Target for Clear Cell Renal Cell Carcinoma

**DOI:** 10.1155/ijog/3645641

**Published:** 2025-01-21

**Authors:** Yanhong Hao, Hongchun Lv, Xu Yan, Yanyan Liang, Aimin Jiang, Yuxia Zhao

**Affiliations:** ^1^Department of Medicine, Xinyang Vocational and Technical College, Xinyang, Henan, China; ^2^Department of Urology, Changhai Hospital, Naval Medical University (Second Military Medical University), Shanghai, China

**Keywords:** clear cell renal cell carcinoma, exportin, pan-cancer, single-cell sequence, XPO1

## Abstract

**Background:** Recently, exportin gene family members have been demonstrated to play essential roles in tumor progression. However, research on the clinical significance of exportin gene family members is limited in clear cell renal cell carcinoma (ccRCC).

**Methods:** Pan-cancer data, ccRCC multiomics data, and single-cell sequence were included to analyze the differences in DNA methylation modification, single nucleotide variations (SNVs), copy number variations (CNVs), and expression levels of exportin gene family members. Non-negative matrix factorization was used to identify molecular subtypes based on exportin gene family members, and the prognostic and biological differences of different molecular subtypes were compared across multiple dimensions.

**Results:** Exportin gene family members were upregulated in pan-cancer expression, and their aberrant expression was significantly influenced by DNA methylation, SNV, and CNV, particularly in ccRCC. Based on the expression matrix of exportin gene family members, two molecular subtypes, exportin famliy genes (XPO)–based subtype 1 (XPS1) and exportin famliy genes (XPO)–based subtype 2 (XPS2), were identified. The expression levels of exportin gene family members in the XPS2 subtype were significantly higher than those in XPS1, and the prognosis was poorer. The XPS2 subtype had lower immune component abundance and higher immune exhaustion scores. Its response rate to immunotherapy was significantly lower than that of the XPS1 subtype, but it was more sensitive to small molecules, including mercaptopurine and nutlin. Among them, exportin-1 (XPO1) is a potential diagnostic and therapeutic target for ccRCC, which can promote renal cancer progression by activating the PI3K-AKT-mTOR (phosphatidylinositol 3-kinase (PI3K)/AKT serine/threonine kinase (AKT)/mechanistic target of rapamycin (MTOR)) and interferon alpha pathways.

**Conclusion:** This study analyzed the variations of exportin gene family members at the pan-cancer level and identified two distinct ccRCC subtypes, which can guide personalized management of patients.

## 1. Introduction

Renal cell carcinoma (RCC) is a prevalent urological malignancy, with clear cell renal cell carcinoma (ccRCC), also known as kidney renal clear cell carcinoma (KIRC), being the most common subtype, accounting for approximately 75% of all RCC cases [1, 2]. Despite advancements in diagnostic techniques and treatment strategies, ccRCC remains a significant challenge due to its high metastatic potential and resistance to conventional therapies, such as chemotherapy and radiation [3]. The 5-year survival rate for patients with advanced or metastatic ccRCC remains poor, underscoring the need for more effective therapeutic approaches [4]. Recently, targeted therapies and immunotherapy have emerged as promising treatment options for ccRCC. Targeted therapies, such as tyrosine kinase inhibitors (TKIs) and mammalian target of rapamycin (mTOR) inhibitors, have improved patient outcomes by targeting specific molecular pathways involved in tumor growth and angiogenesis [5, 6]. Additionally, immune checkpoint inhibitors (ICIs), such as anti-PD1 and anti-CTLA4 antibodies, have demonstrated remarkable efficacy in a subset of ccRCC patients by harnessing the power of the immune system to combat cancer [7, 8]. However, despite these advancements, the prognosis for patients with advanced ccRCC remains unsatisfactory, emphasizing the need for the identification of novel therapeutic targets and strategies [9–11]. Consequently, it is essential to identify different risk subtypes in KIRC to guide personalized treatment for patients [12].

Exportin proteins, a family of nuclear export receptors, play a crucial role in the nucleocytoplasmic transport of various macromolecules, including proteins and RNAs [13]. These proteins are responsible for maintaining the proper subcellular localization of their cargo, which is essential for normal cellular function. Dysregulation of exportin proteins has been implicated in the development and progression of various cancers, as well as in the acquisition of drug resistance [14]. Therefore, understanding the role of exportin proteins in cancer biology has become an area of intense research interest. Among the exportin family members, exportin-1 (XPO1), also known as Chromosomal Maintenance 1 (CRM1), has emerged as a key player in oncogenesis [13]. XPO1 is responsible for the nuclear export of numerous tumor suppressor proteins, such as p53, p21, and FOXO, as well as other critical molecular players involved in cell cycle regulation, apoptosis, and DNA damage response [15]. Overexpression of XPO1 has been observed in various cancers, including ccRCC, and has been associated with poor prognosis and drug resistance [16]. Consequently, XPO1 has garnered attention as a potential therapeutic target in cancer treatment. Despite the growing evidence implicating exportin proteins, particularly XPO1, in cancer biology, their role in ccRCC remains underexplored. Limited studies have focused on the comprehensive analysis of exportin family members in the context of renal cancer, leaving a significant gap in our understanding of their potential as therapeutic targets. Moreover, the molecular mechanisms underlying the oncogenic functions of XPO1 and exportin family members in ccRCC have not been fully elucidated, hindering the development of effective targeted therapies.

In this study, we systematically analyzed the role of exportin family members in pan-cancer, particularly in KIRC, by integrating multiomics and single-cell data. Through clustering analysis and independent external data, we constructed an exportin family member–based KIRC classification system, termed exportin-based subtype (XPS), and compared it across multiple omics dimensions. XPS can robustly stratify KIRC risk, explain tumor heterogeneity in KIRC patients, and guide personalized drug treatment. Furthermore, we analyzed the prognostic value and biological functions of XPO1 in KIRC. In summary, our study provides new insights for the precise diagnosis and treatment of KIRC by leveraging the characteristics of exportin family members.

## 2. Methods and Materials

### 2.1. Data Collection and Processing

Gene expression matrix data for normal and tumor tissues in the pan-cancer dataset were downloaded from The Cancer Genome Atlas-Genomic Data Commons (TCGA-GDC) database [17]. Corresponding clinical and prognostic information was obtained from the cBioPortal database. The specific tumor types and corresponding sample sizes are summarized in Supporting Information 7: Table S1. All transcriptome sequencing data were mapped using GENCODE27. Additionally, we used the GSE22541, RECA-EU, and ccRCC datasets collected by our center as external validation sets, termed chromophobe renal cell carcinoma (CH-RCC), to evaluate the reproducibility of the subtype classification results, and the detailed baseline information was summarized in Supporting Information 8: Table S2. The expression matrix of GSE22541 was annotated using the AnnoProbe package. If multiple probes corresponded to the same gene, we retained the maximum value for subsequent analysis.

In the analysis of DNA methylation data, we specifically focused on probes located within CpG islands associated with promoter regions. For genes with multiple probes mapped to the same promoter, the median *β*-value across these probes was selected for further analysis. Regarding gene mutations, we included only samples with nonsynonymous variants in the mutation matrix. These variants encompassed missense, nonsense, nonstop mutations, frameshift insertions/deletions, in-frame insertions/deletions, and alterations at splice sites or translation start sites. For copy number alterations (CNAs), genomic segments were aggregated and processed in accordance with methods detailed in prior studies [18–20].

### 2.2. Consensus Clustering

In The Cancer Genome Atlas-kidney renal clear cell carcinoma (TCGA-KIRC) cohort, after obtaining the expression matrix of the exportin family genes (XPO), containing XPO1, CSE1L, XPOT, XPO4, XPO5, XPO6, and XPO7, we performed unsupervised clustering analysis using the ConsensusClusterPlus (Version 1.62.0) package with the following parameters: iterations = 100, possible cluster numbers = 2‐9, cluster algorithm = *K*‐means, and Euclidean distance [21]. We used three indicators to determine the optimal number of clusters: the proportion of ambiguous clustering (PAC) score, the cumulative distribution function (CDF) curve, and the consensus score matrix. Then, we used nearest template prediction (NTP) to recluster the samples in the three previously mentioned validation cohorts and confirmed the reliability of the subtype classification through prognostic analysis after grouping [22].

### 2.3. Differential Expression Analysis, Enrichment Analysis, and Immune Infiltration Analysis

After obtaining the optimal classification, we performed differential expression analysis between the XPO-based subtype 1 (XPS1) and XPO-based subtype 2 (XPS2) subgroups using DESeq2 (Version 1.38.3) [23]. The criteria for identifying differentially expressed genes were as follows: absolute log2FoldChange > 1.5 and adjusted *p* value < 0.05. We used pathways collected in the IOBR (Version 0.99.9) and MSigDB (Version 1.6.0) R packages to perform single-sample gene set enrichment analysis (ssGSEA) between the two subgroups and used the limma package to analyze differences in enrichment scores of relevant pathways between the groups [24–26]. Similarly, we used seven classic immune infiltration scoring algorithms to compare differences in immune cell infiltration between the two groups, including CIBERSORT, EPIC, ESTIMATE, MCP-counter, quanTIseq, TIMER, and xCell (Version 1.1.0) [27–29]. Finally, we used the tumor immune dysfunction and exclusion (TIDE) algorithm to compare the differences in sensitivity to ICI treatment between the subgroups.

### 2.4. Genomic Variation and Drug Sensitivity Analysis

We used the Maftools (Version 2.14.0) package to quantify and visualize the single nucleotide variation (SNV) frequencies of top genes and XPO in the pan-cancer and ccRCC cohorts [30]. Copy number variations (CNVs) were analyzed using the GISTIC 2.0 algorithm, including the percentage of genome alteration (FGA), the fraction of genomic gain (FGG), and the fraction of genome loss (FGL) [31]. FGA represents the proportion of the genome affected by CNAs, while FGG and FGL indicate the fractions of the genome with copy number gains and losses, respectively. Additionally, we used the Genomics of Drug Sensitivity in Cancer (GDSC) database and Connectivity Map (CMAP) to analyze the drug sensitivity of different subgroups [32]. The half-maximal inhibitory concentration (IC50) was quantified using the pRRophetic (Version 0.5) R package. A higher absolute IC50 value indicates lower drug sensitivity for the corresponding tumor subtype [33].

### 2.5. Analysis of Single-Cell Sequence for ccRCC

We used the Seurat (Version 4.3.0.1) package to annotate and process single-cell sequence and spatial transcriptomics data obtained using the 10× Genomics platform. The specific filtering criteria were to remove spots with fewer than 200 detected genes and genes with fewer than 10 read counts. We normalized the single-cell spatial transcriptomics data using the LogNormalize function and performed dimensionality reduction analysis using the Top 30 principal components. We also used the BayeSpace (Version 1.16.0) package to quantify gene expression levels in the single-cell spatial transcriptomics data. We annotated the subpopulations of the single-cell transcriptomes using classic cellular markers and identified the characteristic genes of each subpopulation with the FindAllMarkers function. Additionally, we employed five scoring methods, including AUCell, UCell, singscore, gene set variation analysis (GSVA), and AddModuleScore, to calculate the exportin family score. The average of these five scores was used as the final exportin score, referred to as the XPO score. Based on the median value of the XPO score, macrophages were classified into the XPO high and XPO low score groups. Finally, we analyzed the communication patterns among different cell subpopulations using two widely recognized cell–cell communication R packages containing CellCall (Version 1.0.7) and CellChat (Version 1.0.7). For annotating the spatial transcriptomics data, we referred to previous single-cell transcriptomics studies and utilized the AddModuleScore function in the Seurat package.

### 2.6. Statistical Analysis

All statistical tests and visualizations in this study were performed using R and SPSS software. For continuous variables, we used Student's *t*-test or Wilcoxon rank-sum test for intergroup comparisons. The chi-square test and Fisher's exact test were used for statistical testing of categorical variables. Spearman's correlation coefficient was used for correlation analysis. We used the survminer and survival R packages for Kaplan–Meier and Cox regression analyses. The false discovery rate (FDR) was calculated using the Benjamini–Hochberg adjustment. In this study, *p* < 0.05 or FDR < 0.05 was considered statistically significant. More analysis information could refer to our previous works [34–37].

## 3. Results

### 3.1. Heterogeneous Expression Levels of XPO in Pan-Cancer Are Influenced by DNA Methylation and Genomic Variations

**Figure 1:**
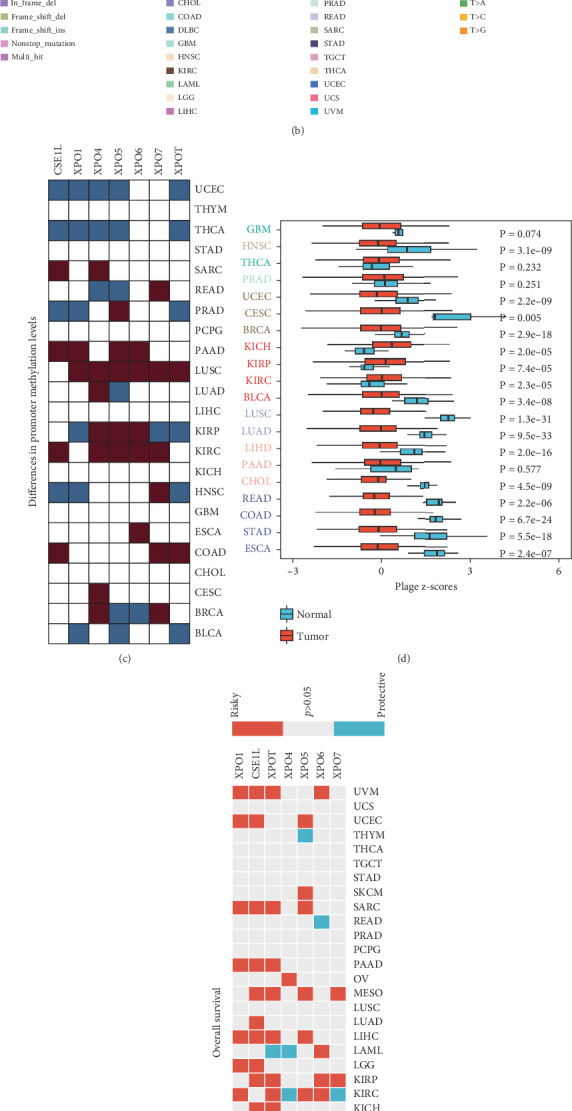
Expression differences, mutation frequencies, and DNA methylation levels of exportin family genes across pan-cancer. (a) Expression differences of exportin family genes between tumor and adjacent tissues across various cancer types (pan-cancer). Red represents higher expression in tumor tissues, while blue represents lower expression. (b) Oncoplot showing the mutation distribution of exportin family genes across pan-cancer. (c) Differential methylation levels of exportin family genes at their promoter regions. (d) Boxplot displaying the variation in exportin family gene-related scores across pan-cancer. (e) Prognostic value of exportin family genes in pan-cancer. Red indicates a risk factor, while blue indicates a protective factor. (f) Pie plot illustrating the copy number variations (CNVs) of exportin family genes among different cancer types. Red represents gene amplification, while blue represents gene deletion.

We first analyzed the different targeting patterns of XPO at the pan-cancer level and the potential regulatory mechanisms causing this abnormality. Using two-box analysis of The Cancer Genome Atlas (TCGA) and Genotype-Tissue Expression (GTEx) databases, we compared the expression levels of XPO, including XPO1, CSE1L, XPOT, XPO5, and XPO6, in each tumor and normal tissue. We found that the majority of XPO were significantly upregulated in tumors, while XPO4 and XPO7 were downregulated in some cancer types (Figure 1(a)). Next, we analyzed the SNVs of XPO in the TCGA pan-cancer cohort and observed that almost all XPO had a high frequency of SNVs, greater than 18% (Figure 1(b)). Methylation plays a crucial role in regulating gene expression levels; typically, higher methylation suppresses gene expression, but in certain situations, it can also promote gene expression. We observed heterogeneity in the promoter region methylation of XPO across different cancer types. Multiple XPO exhibited hypermethylation in the promoter regions in KIRC, kidney renal papillary cell carcinoma (KIRP), lung squamous cell carcinoma (LUSC), and pancreatic adenocarcinoma (PAAD), while hypomethylation was observed in thyroid carcinoma (THCA) and uterine corpus endometrial carcinoma (UCEC) (Figure 1(c)). These results may partly explain the abnormal expression levels of XPO in tumors.

Considering the oncogenic effects of XPO, we calculated the exportin-related scores in each cancer type and corresponding normal tissues using the GSVA algorithm. We found that the exportin signal score was significantly upregulated only in the tumor tissues of kidney chromophobe (KICH), KIRP, and KIRC, while it was significantly downregulated in other tumors (Figure 1(d)). Furthermore, we evaluated the impact of the exportin signal on tumor patient prognosis, focusing on four prognostic indicators in the TCGA pan-cancer data: overall survival (OS), progression-free interval, disease-specific survival, and disease-free interval. The results showed that the exportin signal had a consistent prognostic effect in most tumors, with higher expression levels indicating a worse prognosis. This phenomenon was particularly significant in uveal melanoma (UVM), UCEC, sarcoma (SARC), PAAD, mesothelioma (MESO), liver hepatocellular carcinoma (LIHC), KIRP, KIRC, and bladder urothelial carcinoma (BLCA) (Figure 1(e) and Supporting Information 1: Figures S1(A–C)). Finally, we analyzed the overall landscape of CNV in the exportin gene family. The results indicated that most exportin genes had significant amplification events in pan-cancer, with CSE1L being particularly prominent (Figure 1(f)). These findings suggest that events such as methylation modification, CNV, and SNV can affect the abnormalities of the exportin gene family in pan-cancer, thereby impacting tumor patient prognosis, especially in renal cancer.

### 3.2. Expression Matrix of XPO Gene Family Distinguishes ccRCC Into Subgroups With Different Risk Stratifications

**Figure 2:**
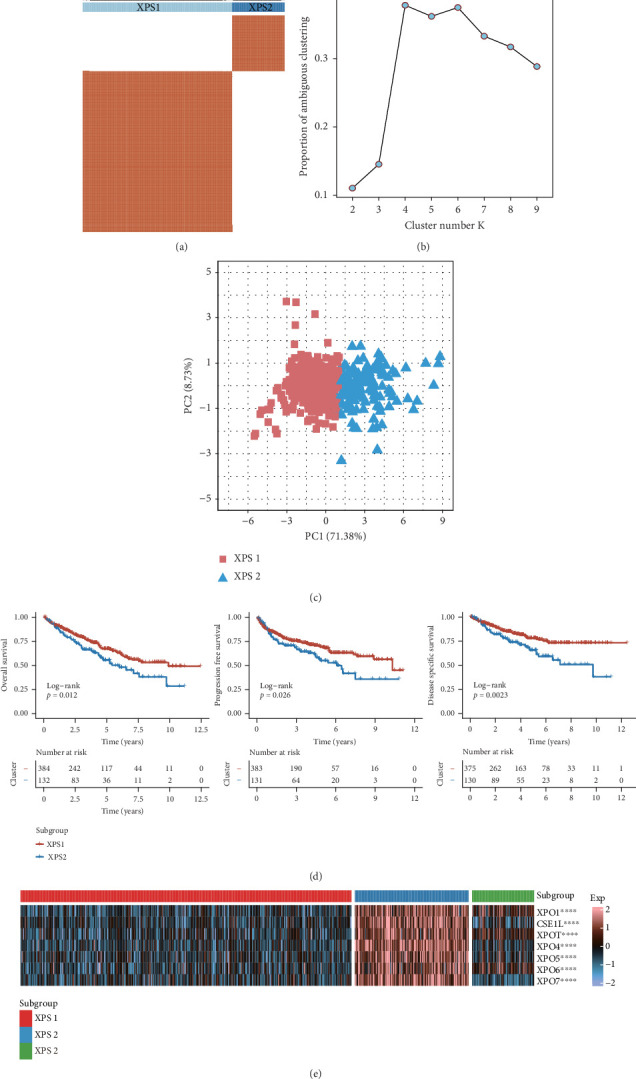
Classification of KIRC patients into two subtypes based on the expression of exportin family genes. (a) Consensus score matrix based on the expression of exportin family genes in TCGA-KIRC samples when the number of clusters (*k*) is set to 2. (b) PAC (partition around medoids) analysis for determining the optimal number of clusters, ranging from 2 to 9. (c) Principal component analysis (PCA) plot showing the two-dimensional distribution of XPS1 and XPS2 subtypes. (d) Kaplan−Meier curves for overall survival, progression-free survival, and disease-specific survival in the TCGA-KIRC cohort. (e) Heatmap displaying the relative expression differences of exportin family genes among XPS1, XPS2, and normal tissue samples. ⁣^∗∗∗∗^*p* < 0.0001.

Given the important role of the exportin gene family in renal cancer prognosis, with ccRCC being the most common pathological subtype, we performed unsupervised clustering using the expression matrix of the exportin family to better understand its role in ccRCC progression. Combining the consensus matrix, delta area plot for *K*, and principal component analysis (PCA), we determined the optimal number of clusters to be two, defined as XPS1 and XPS2 (Figures 2(a), 2(b), and 2(c)). The two patient groups had significant prognostic differences, as indicated by the Kaplan–Meier curves for OS, progression-free survival, and disease-specific survival (Figure 2(d) and Supporting Information 9: Table S3). We also used the classic NTP algorithm to validate the robustness of the clustering results based on the exportin gene family in other ccRCC cohorts. We observed that the XPS2 subtype had a worse prognosis in three independent datasets: GSE22541, CH-RCC, and RECA-EU (Supporting Information 2: Figures S2(A–F)). Additionally, we observed higher expression levels of XPO in XPS2 (Figure 2(e)).

### 3.3. XPS1 and XPS2 Exhibit Extensive Biological and Metabolic Heterogeneity

**Figure 3:**
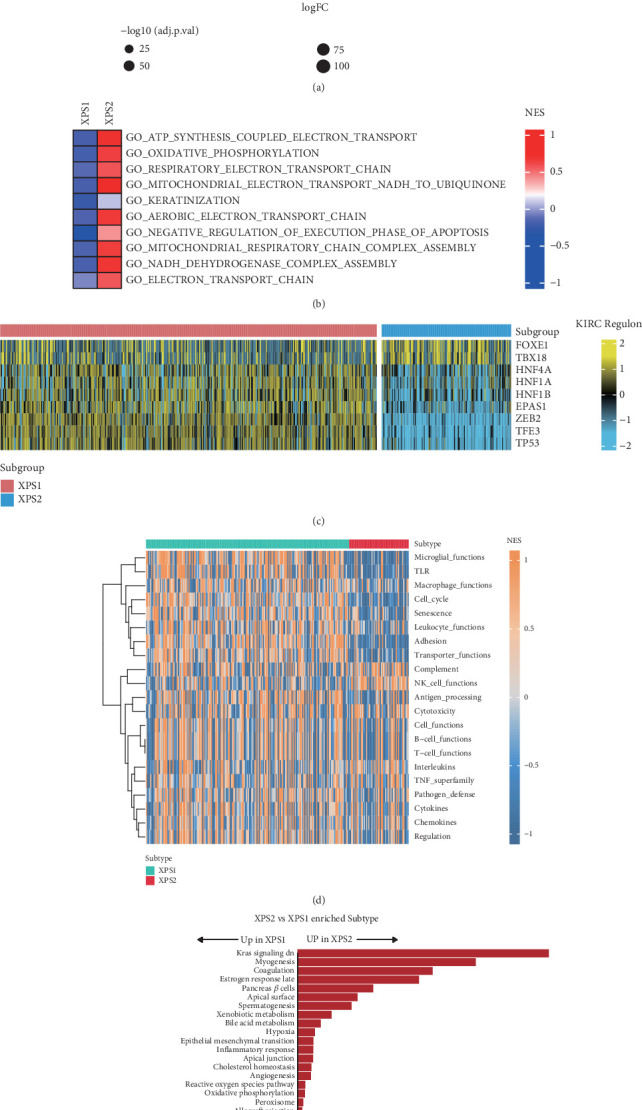
Distinct biological characteristics between XPS1 and XPS2 subtypes. (a) Heatmap showing differentially expressed genes between XPS1 and XPS2 subtypes. (b) Heatmap illustrating the enriched Gene Ontology (GO) pathways between XPS1 and XPS2 subtypes. (c) Heatmap depicting the differences in KIRC-related transcription factor activity between XPS1 and XPS2 subtypes. (d) Heatmap displaying the differences in immune-related pathways between XPS1 and XPS2 subtypes. (e) Bar plot comparing the activation of hallmark gene sets between the two subtypes.

To investigate the biological distinctions between the two subtypes, we performed differential expression and gene set enrichment analyses. Relative to the XPS1 subtype, which exhibited a more favorable prognosis, the XPS2 subtype was characterized by upregulation of genes such as RHCG, PVALB, KLK1, and CGA (Figure 3(a)). Gene set enrichment analysis revealed significant activation of pathways related to adenosine triphosphate (ATP) metabolism, oxidative phosphorylation, electron transport chain, and mitochondrial metabolism in the XPS2 subtype (Figure 3(b)). Given the crucial role of transcription factors in driving tumor progression, we identified FOXE1 and TBX18 as being relatively more active in the XPS2 subtype, whereas HNF4A, HNF1A, HNF1B, EPAS1, CEB2, TFE3, and TP53 displayed higher activity in the XPS1 subtype (Figure 3(c)). Notably, gene set enrichment analysis demonstrated that most immune-related pathways were significantly downregulated in the XPS2 subtype compared to XPS1, with the exception of the natural killer (NK) cell pathway, which exhibited relative activation (Figure 3(d)). Hallmark gene set analysis indicated the activation of classic oncogenic pathways, including Kras signaling, myogenesis, hypoxia, and epithelial-mesenchymal transition, in the XPS2 subtype. In contrast, cell cycle–related pathways, such as the G2M checkpoint, Myc targets, and E2F targets, were more active in the XPS1 subtype (Figure 3(e)). Interestingly, we observed striking metabolic heterogeneity between the XPS1 and XPS2 subtypes. While the XPS2 subtype displayed upregulation of a limited number of metabolic pathways, such as linoleic acid metabolism and cardiolipin biosynthesis, the majority of metabolic pathways were more active in the XPS1 subtype (Supporting Information 3: Figure S3(A)). Consistently, only cancer cell, chemokine, cytokine, and regulatory T cell (Treg) pathways were activated in the XPS2 subtype, whereas most tumor immune-related pathways were relatively suppressed (Supporting Information 3: Figure S3(B)). These findings underscore the significant metabolic and immunological heterogeneity that exists between distinct exportin-defined subtypes of ccRCC.

### 3.4. XPS2 Subtype Exhibits an Immune Exclusion Phenotype

**Figure 4:**
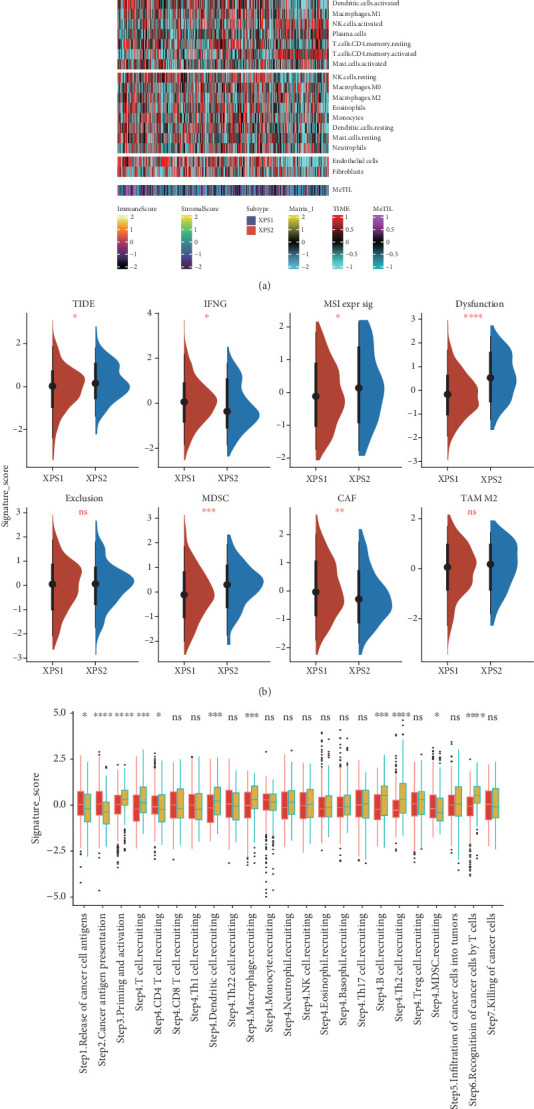
Differences in immune infiltration between XPS1 and XPS2 subgroups. (a) Heatmap showing immune scores, stromal scores, expression levels of immune checkpoint inhibitor (ICI)–related molecules, and the abundance of infiltrating immune cells in different subgroups. (b) Violin plot illustrating the differences in scores related to the tumor immune dysfunction and exclusion (TIDE) algorithm between the two subgroups. (c) Boxplot comparing the immune cycle scores obtained using the tumor immunophenotype (TIP) algorithm between the XPS1 and XPS2 groups. ⁣^∗^*p* < 0.05, ⁣^∗∗^*p* < 0.01, and ⁣^∗∗∗^*p* < 0.001; ns: no significance.

Immunotherapy is the preferred treatment option for advanced renal cancer. We further explored the differences in the immune profiles between the two ccRCC subtypes. We observed higher expression levels of PDCD1 and CD247 in the XPS2 subtype compared to XPS1, while the latter had higher expression levels of CD274 and TNFRSF9 (Figure 4(a)). Based on deconvolution analysis, we found significant differences in immune infiltration between the two molecular subtypes, with higher proportions of Treg and CD4 memory-activated cells in XPS2 and an increased proportion of endothelial cells in XPS1 (Figure 4(a)). Using classic deconvolution algorithms, including TIMER, CIBERSORT, quanTIseq, MCP-counter, xCell, and EPIC, we found that XPS2 exhibited an immune exclusion phenotype, characterized by lower scores for most immune cell types, while T cell, NK cell, and Treg infiltration abundances were higher (Supporting Information 4: Figure S4(A)). Additionally, we systematically analyzed the expression level differences of chemokines, chemokine receptors, major histocompatibility complex (MHC), immune inhibitors, and immune stimulators between the two subtypes and found lower expression levels of tumor immune-related secretory factors in XPS2 (Supporting Information 4: Figure S4(B)).

Furthermore, using the TIDE algorithm, we identified higher TIDE, microsatellite instability (MSI), antitumor dysfunction, and myeloid-derived suppressor cell (MDSC) scores in XPS2 (Figure 4(b)). After calculating the antitumor immune cycle scores of tumor immunity in the microenvironment (TIME), we found that the overall score was higher in XPS1, while XPS2 had higher scores for priming and activation, dendritic cell (DC), macrophage, B cell, T helper 2 (Th2) cell recruiting, and recognition of cancer cells by T cells compared to the XPS1 subgroup (Figure 4(c)).

### 3.5. XPS Typing Can Guide Precision Medicine for ccRCC Patients

**Figure 5:**
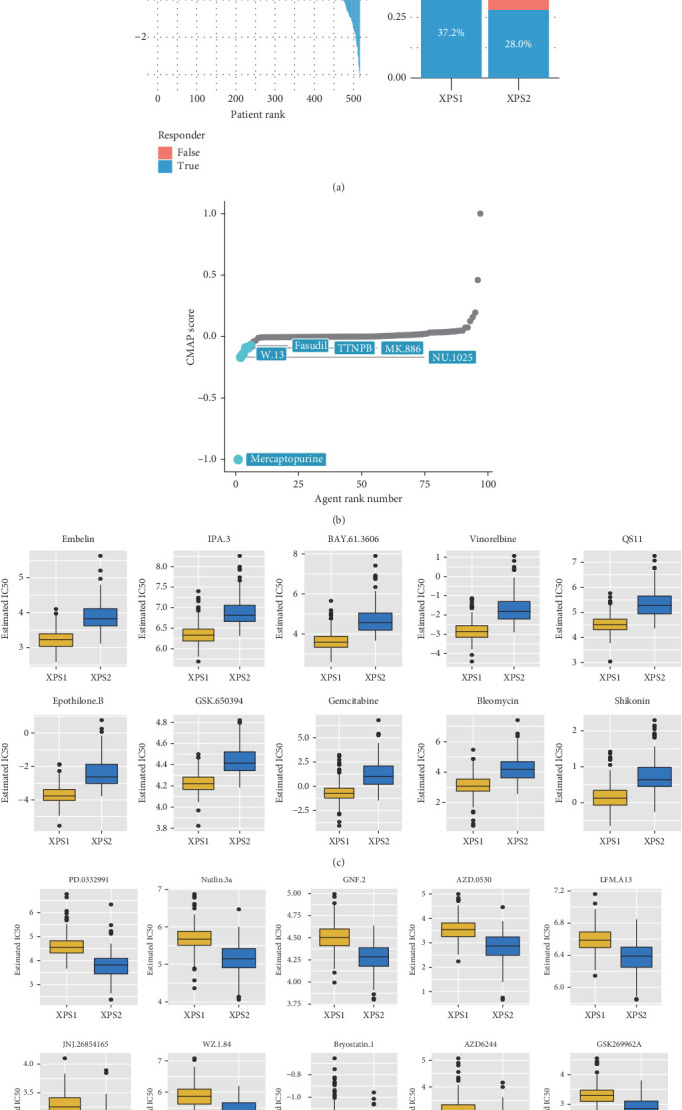
XPS subtyping is closely associated with KIRC treatment response rates. (a) TIDE algorithm analysis comparing the immunotherapy response rates between XPS1 and XPS2 subgroups. (b) Scatter plot showing the Connectivity Map (CMAP) scores of different drugs in the XPS2 subgroup. Higher CMAP scores indicate increased sensitivity of the XPS2 subgroup to the corresponding drug. (c) Boxplot displaying the sensitive drugs for XPS1 treatment and (d) XPS2 treatment based on an analysis of the Genomics of Drug Sensitivity in Cancer (GDSC) database.

We compared the sensitivity of the two patient groups to immunotherapy and targeted therapy. First, using the TIDE algorithm, we observed that the response rate to ICI treatment was significantly lower in XPS2 compared to XPS1 (28% in XPS2 vs. 37.2% in XPS1) (Figure 5(a)). Furthermore, we compared the sensitivity of different subtypes to targeted drugs and found that XPS2 was resistant to six classic targeted drugs, including axitinib, crizotinib, imatinib, pazopanib, temsirolimus, and sunitinib, while being more sensitive to dasatinib, lisitinib, and gefitinib (Supporting Information 5: Figure S5). Based on the CMAP database, we identified potential therapeutic drugs for the XPS2 subtype with a worse prognosis, including mercaptopurine, W13, fasudil, and TTNPB (tigilanol tiglate naphthalenone phenyl-B), which could be potential therapeutic targets for the XPS2 subtype with a worse prognosis (Figure 5(b)). Additionally, using IC50 data of different cell lines to small molecule inhibitors in the Cancer Cell Line Encyclopedia (CCLE), we evaluated the sensitivity of different subtypes to other drugs. The results suggested that embelin, IPA3, BAY-61-3606, vinorelbine, and QS11 were effective therapeutic targets for the XPS1 subtype, while PD-0332991, nutlin-3a, GNF2, AZD-0530, and LFM-A13 were effective targets for the XPS2 subtype (Figures 5(c) and 5(d)).

### 3.6. XPS1 and XPS2 Have Distinct Mutation Characteristics

**Figure 6:**
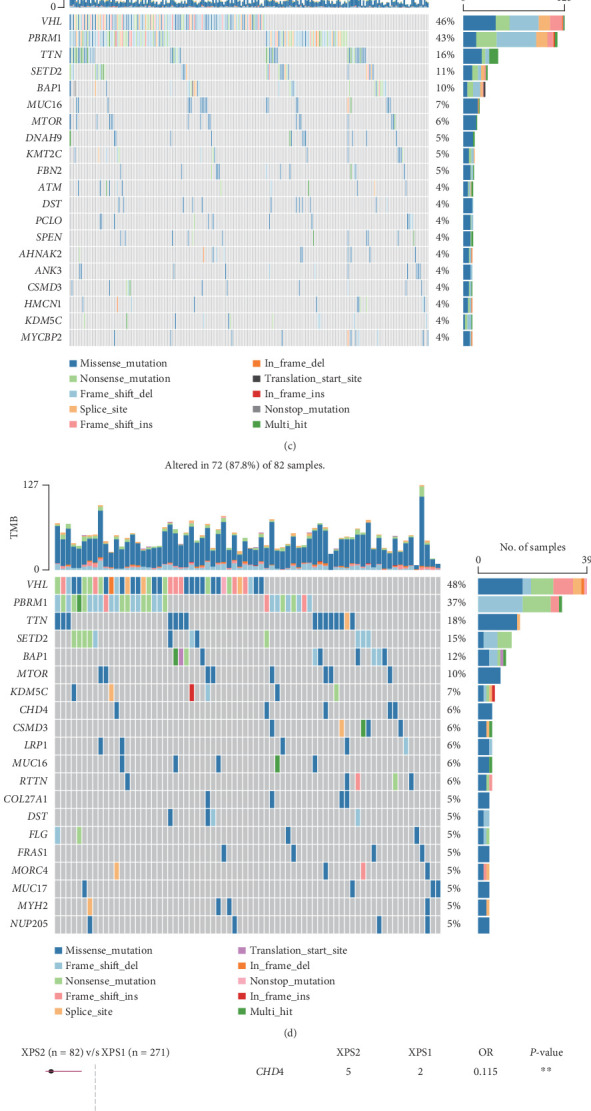
Genomic mutation profiles of the two subgroups. (a) Waterfall plot showing the overall mutation rate of exportin family genes in the TCGA-KIRC cohort. (b) Waterfall plot illustrating the overall profile of frequently mutated genes in the TCGA-KIRC cohort. (c) Waterfall plot comparing the mutation frequency of frequently mutated genes between the XPS1 and (d) XPS2 subgroups. (e) Forest plot displaying the prognostic value of mutated genes between the two subgroups. (f) Bar plot comparing the differences in copy number variations (CNVs) between the two subgroups. ⁣^∗^*p* < 0.05 and ⁣^∗∗^*p* < 0.01.

Overall, we found that the mutation frequency of XPO in ccRCC was relatively low, with XPO7 having a relatively higher mutation frequency (Figure 6(a)). We found that classic renal cancer–related genes such as VHL, PBRM1, TTN, and SETD2 had higher mutation frequencies in the TCGA-KIRC cohort, with PBRM1 having a higher mutation rate in XPS1 compared to XPS2 (43% vs. 37%), while mTOR had a lower mutation rate in XPS1 compared to XPS2 (6% vs. 10%) (Figures 6(b), 6(c), and 6(d)). Based on prognosis-related analysis, we found that CHD4 and RTTN were protective mutations in the XPS2 subtype (Figure 6(e)). Overall, we found that the CNA frequency in the XPS2 subtype, including gain and loss frequencies, was higher than that in the XPS1 subtype (Figure 6(f)).

### 3.7. XPO1 Is a Potential Biomarker for ccRCC

**Figure 7:**
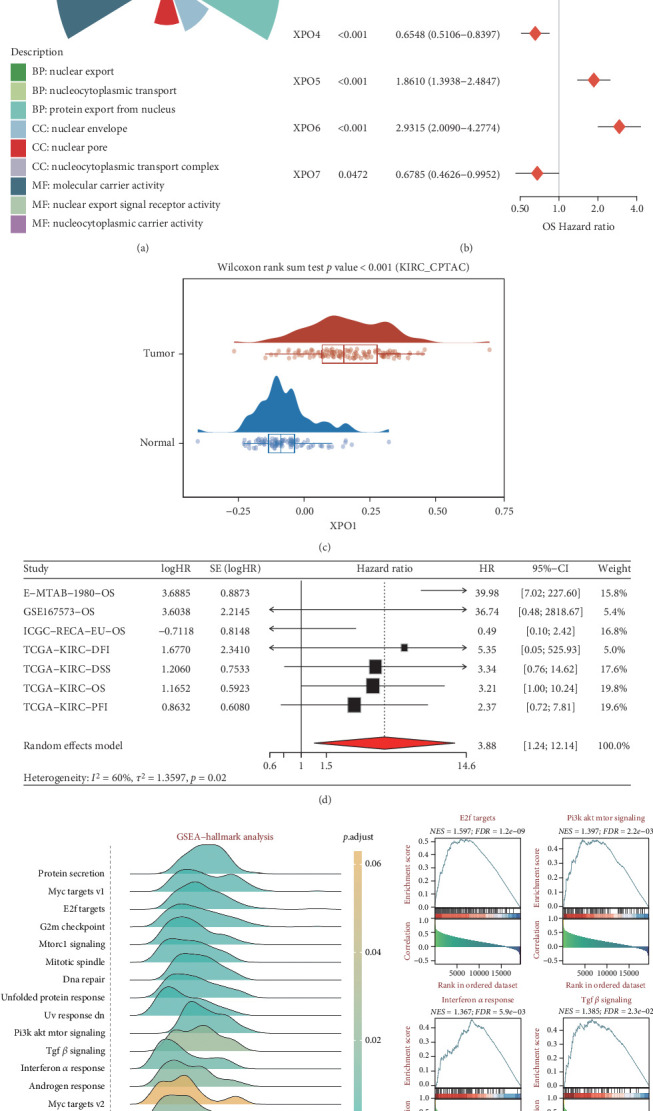
Prognostic and biological roles of exportin family genes in KIRC. (a) Functional annotation of exportin family genes. (b) Univariate regression analysis assessing the impact of exportin family genes on the prognosis of KIRC patients. (c) Expression differences of XPO1 between KIRC tumors and adjacent tissues. (d) Meta-analysis evaluating the comprehensive prognostic effect of XPO1 in KIRC. (e) Determining the biological role of XPO1 in KIRC based on correlation analysis.

We first studied the molecular mechanisms of the exportin family in ccRCC. Gene Ontology (GO) analysis suggested that the exportin family is mainly involved in biological processes related to nuclear export (Figure 7(a)). Prognostic analysis indicated that XPO1, XPOT, XPO5, and XPO6 were risk factors for ccRCC patient prognosis, while XPO4 and XPO7 were protective factors (Figure 7(b)). Considering that XPO1 is the most widely studied target in the exportin family, we focused on XPO1. We observed that XPO1 expression levels were significantly upregulated in ccRCC tumor tissues (Figure 7(c)). Integrated prognostic analysis further suggested that XPO1 is involved in the progression of ccRCC patients (Figure 7(d)). Through similarity analysis and enrichment analysis, we observed that XPO1 could participate in multiple tumor progression signals in ccRCC, including E2F, PI3K-AKT, interferon-alpha, and TGF-beta signaling axes (Figure 7(e)).

### 3.8. Decoding the Role of Exportin Family at Single-Cell Level

**Figure 8:**
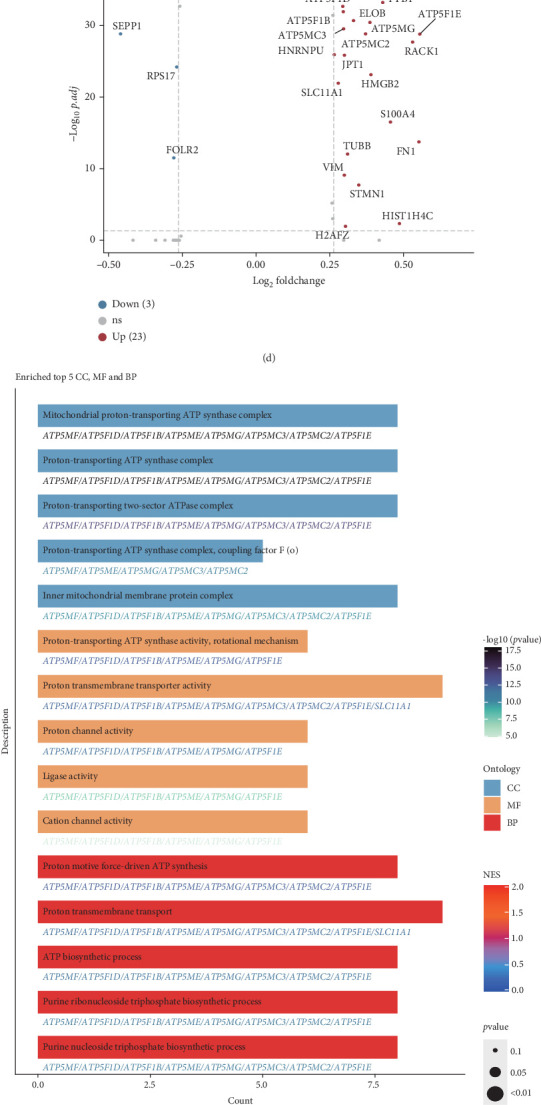
Single-cell subgroup distribution and functional differences of macrophages with varying exportin family scores. (a) t-SNE plot showing the distribution and proportions of each cell population. (b) Dot plot showing the marker genes of each cell population. (c) Dot plot showing the exportin family scores for each cell population calculated using five different algorithms. (d) Volcano plot showing differentially expressed genes between XPO high macrophages and XPO low macrophages. (e) Bar plot displaying enriched terms derived from the differential genes. (f) Chord diagram illustrating the communication strength between cell populations based on CellCall analysis. (g) Dot plot showing cell–cell communication enrichment scores related to tumor-associated pathways derived from CellCall analysis.

To better understand the role of the exportin family in ccRCC and its microenvironment, we performed further analyses using single-cell transcriptomic data from both primary ccRCC and local metastases (tumor thrombus). Using classical single-cell markers, we identified a total of 48,235 high-quality single cells, encompassing B cells, endothelial cells, fibroblasts, macrophages, mast cells, monocytes, neutrophils, NK cells, proliferating cells, proximal tubule (PT) epithelial cells, T cells, and tumor epithelial cells (Figures 8(a) and 8(b)). We calculated the exportin family score and observed that this score was elevated in macrophages and proliferating cells (Figure 8(c)). Considering the critical role of macrophages in tumor progression, we divided macrophages into XPO high and XPO low macrophage subgroups based on the exportin family score. Differential expression analysis revealed that metabolic-related genes such as ATP5F1E, ATP5MG, RACK1, and HMGB2 were upregulated in XPO high macrophages (Figure 8(d)). Enrichment analysis further indicated significant enrichment of energy metabolism pathways, including the ATP synthase complex and adenosine triphosphatase (ATPase) complex (Figure 8(e)). Additionally, GSVA suggested that XPO high macrophages were primarily involved in canonical protumor pathways such as hypoxia, IL6-JAK-STAT3, and PI3K-AKT-mTOR (phosphatidylinositol 3-kinase (PI3K)/AKT serine/threonine kinase (AKT)/mechanistic target of rapamycin) signaling (Supporting Information 6: Figure S6(A)). Given that intercellular interactions play a significant role in tumor progression, we employed CellCall to analyze cell–cell communication. We found that XPO high macrophages exhibited more frequent interactions with other cell types compared to XPO low macrophages (Figure 8(f)). Among these interactions, XPO high macrophages and endothelial cells were engaged in pathways associated with RCC, TNF signaling, and mTOR signaling—pathways classically linked to ccRCC (Figure 8(g)). Further analysis with CellChat revealed that the CD72-APP signaling pathway between XPO high macrophages and endothelial cells contributed to ccRCC progression (Supporting Information 6: Figure S6(B)). CellCall analysis also identified systemic interactions between macrophages and endothelial cells, involving pathways such as SERPIN1-LRP6, VEGFA/VEGFB-KDR/FLT1, and PDGFC-FLT1/FLT4/KDR (Supporting Information 6: Figure S6(C)).

**Figure 9:**
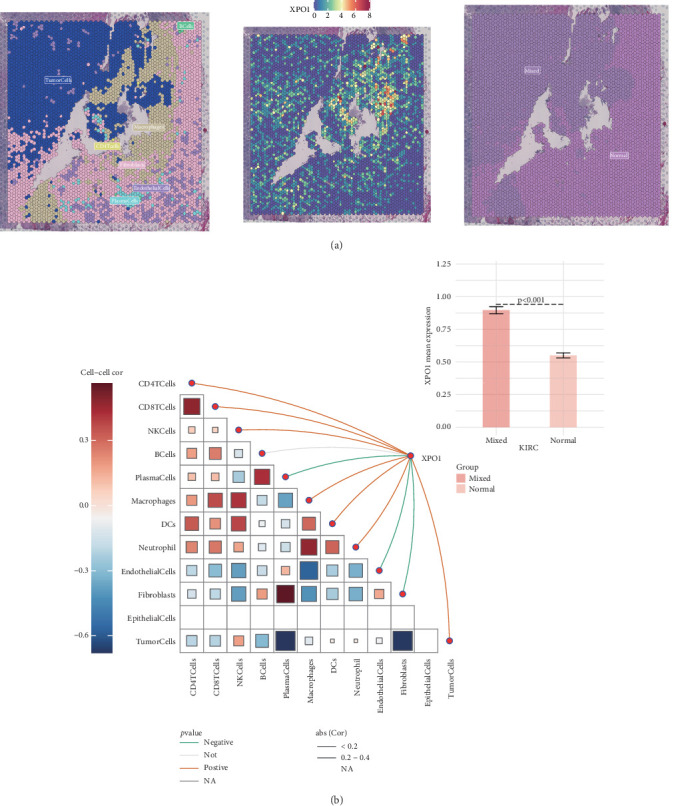
XPO1 is highly expressed in tumor-mixed regions and negatively correlated with stromal component infiltration. (a) Expression level and localization of XPO1 in the KIRC spatial transcriptome. (b) Line plot illustrating the correlation between XPO1 expression levels and the infiltration abundance of other cellular components in the spatial transcriptome.

Next, we analyzed the expression level of XPO1 and its relationship with tumor microenvironment infiltration at the single-cell spatial transcriptome level in ccRCC. XPO1 was mainly expressed in the tumor cell region and had higher expression levels in the infiltration regions of tumor cells and other immune and stromal components (Figure 9(a)). Correlation analysis suggested that XPO1 expression levels were positively correlated with the infiltration levels of multiple immune cells, including CD4 T cells, CD8 T cells, NK cells, macrophages, DCs, and neutrophils, while negatively correlated with plasma cells, endothelial cells, and fibroblasts (Figure 9(b)). These results suggest that XPO1 in tumor cells can influence renal cancer progression by regulating the infiltration of immune and stromal components.

## 4. Discussion

In this study, we performed a comprehensive multiomics analysis of the exportin gene family across pan-cancer datasets, with a particular focus on ccRCC. Our results demonstrated that XPO are frequently dysregulated in various cancer types, with their aberrant expression significantly influenced by DNA methylation, SNV, and CNV. Notably, we identified two distinct molecular subtypes of ccRCC, termed XPS1 and XPS2, based on the expression matrix of exportin genes. The XPS2 subtype, characterized by higher exportin gene expression, exhibited a worse prognosis and an immune exclusion phenotype, along with a lower response rate to immunotherapy compared to the XPS1 subtype. Furthermore, we identified XPO1 as a potential diagnostic and therapeutic target for ccRCC, as it was found to be involved in the activation of PI3K-AKT-mTOR and interferon-alpha pathways, thereby promoting renal cancer progression.

Our findings are consistent with previous studies that have reported the dysregulation of exportin genes across various cancer types and their association with patient prognosis. For instance, XPO1 has been identified as a critical player in tumorigenesis, with its overexpression noted in several cancers, including pancreatic, lung, and colorectal cancers [13]. This overexpression is often linked to poor patient prognosis and may be influenced by CNVs in tumor cells. XPO1's role extends beyond mere transport; it also inhibits immune responses, thereby facilitating tumor progression. Specifically, XPO1 affects pathways related to the cell cycle and oxidative phosphorylation while promoting the expression of oncogenes and immune checkpoint genes. It has been shown to influence the activity of immune cells within the tumor microenvironment, particularly CD8+ T cells, which are crucial for antitumor immunity. Furthermore, XPO1's expression correlates with tumor mutational burden (TMB) and MSI, suggesting its involvement in genomic instability that drives cancer progression [16]. Another exportin family member, XPO6, has emerged as a significant factor in various cancers, notably breast cancer. Its upregulation is associated with poor survival outcomes, marking it as a potential oncogenic driver. The mechanism involves the spatial regulation of profilin-1, a protein that acts as a tumor suppressor when localized to the nucleus. By exporting profilin-1 from the nucleus, XPO6 inhibits its function, promoting tumorigenesis. Zhu et al. found that reducing XPO6 levels can trigger antitumor effects by allowing nuclear accumulation of profilin-1, suggesting that targeting XPO6 could enhance the efficacy of existing therapies, such as bromodomain and extraterminal domain (BET) inhibitors like JQ1 [38]. The evidence points towards a nononcogene addiction model where cancer cells become reliant on the dysregulated nuclear export processes mediated by exportins like XPO6. However, to the best of our knowledge, no previous study has systematically investigated the role of the exportin gene family across cancers or performed a reclustering analysis based on the exportin gene expression matrix. Our study addresses this gap by identifying the XPS2 subtype, characterized by relatively higher expression of exportin genes, inferior prognosis, and therapy resistance. These findings suggest that targeting exportin family members, especially XPO1, could be a supplementary therapeutic strategy for ccRCC.

To enhance the clinical applicability of the XPS1 and XPS2 subtypes, we not only validated their reproducibility in local and external cohorts but also provided biological interpretations of the XPS classification system across multiple dimensions. Firstly, through differential analysis and enrichment analysis, we discovered that the XPS2 subtype undergoes extensive metabolic reprogramming, with significant activation of pathways such as oxidative phosphorylation, mitochondrial electron transport NADH to ubiquinone, and the electron transport chain. Additionally, hallmark analysis revealed that the XPS2 subtype exhibited significant activation of Kras and myogenesis pathways, which may contribute to the progression of this subtype. As a classic metabolic disease, renal cancer is characterized by the inactivation of the VHL gene and abnormal activation of the Ras-PI3K-AKT-mTOR pathway, leading to metabolic reprogramming that includes glutamine, tryptophan, and arginine metabolism, thereby promoting the occurrence and development of renal cancer [39]. A recent study by Bezwada et al. employed an in vivo tracing strategy using C13 labeling and found that renal cancer development and metastasis exhibit distinct metabolic characteristics, with mitochondrial electron transport being a key factor influencing renal cancer metastasis [40]. Tian et al. discovered that oxidative phosphorylation is an important feature of advanced renal cancer patients developing resistance or acquired resistance to ICI. Knocking down the Mitochondrial Complex I subunit, Ndufb8, can reduce the exhaustion of intratumoral CD8 T cells, improve their function, and enhance the efficacy of immunotherapy [41]. Consistent with our aforementioned results, the abnormally activated oxidative phosphorylation–related pathways in the XPS2 subtype can induce the inhibition of immune cell infiltration and promote immune exhaustion, ultimately leading to the low response rate to ICIs and clinical progression of this subtype. Interestingly, we found that cardiolipin metabolism was significantly activated in XPS2. Previous research suggests that cardiolipin is vital for the assembly and stability of mitochondrial protein complexes, particularly those involved in oxidative phosphorylation [42]. Alterations in cardiolipin composition can affect the efficiency of the electron transport chain, leading to metabolic reprogramming in cancer cells [43]. Furthermore, linoleic acid metabolism was relatively activated in XPS2. Linoleic acid is essential for membrane biosynthesis, providing the necessary components for rapidly proliferating cancer cells. It contributes to the formation of lipid bilayers and signaling molecules that can enhance cell proliferation and survival under stress conditions [44]. Previous works have revealed that linoleic acid metabolism can modulate the immune microenvironment of tumors. Increased levels of linoleic acid and its metabolites can influence immune cell recruitment and polarization, often leading to an immunosuppressive environment that facilitates tumor growth. For instance, certain metabolites derived from linoleic acid may inhibit T cell activation or promote Treg functions, thereby dampening antitumor immunity [45]. These results suggest that the XPS2 subtype can impact antitumor immune functions through extensive metabolic reprogramming, ultimately leading to its malignant progression and low response to therapy.

In addition, we identified small molecules including PD-0332991, nutlin-3a, and GNF2 as potential complementary therapeutic options for ccRCC by drug sensitivity analysis. Among them, PD-0332991, also known as palbociclib, is a selective inhibitor of cyclin-dependent kinases 4 and 6 (CDK4/6) that plays a significant role in cancer therapy, particularly in regulating the cell cycle. Its primary mechanism involves blocking the phosphorylation of the retinoblastoma (Rb) protein, which is crucial for controlling cell proliferation. By inhibiting CDK4/6, PD-0332991 induces a G1 phase arrest in the cell cycle, preventing cells from progressing to the S phase where DNA replication occurs. This action leads to reduced cell growth and proliferation in various malignancies, including breast cancer and RCC [46–48]. Besides, nutlin-3a is a small-molecule inhibitor of the MDM2 protein, which plays a critical role in regulating the p53 tumor suppressor pathway. By disrupting the interaction between MDM2 and p53, nutlin-3a stabilizes p53, leading to its activation. This mechanism has significant implications for cancer therapy, particularly in tumors that retain wild-type p53. In preclinical studies, nutlin-3a has demonstrated promising results in inhibiting tumor growth and enhancing apoptosis in ccRCC models, highlighting its potential as a therapeutic agent [49, 50]. GNF2, an allosteric inhibitor of the Bcr-Abl fusion protein, is primarily recognized for its application in treating chronic myelogenous leukemia (CML). It operates by binding to the myristate-binding site of c-Abl, which leads to the inhibition of Bcr-Abl activity. This mechanism makes GNF2 a promising candidate in cancer therapy, particularly for cases resistant to conventional treatments. ccRCC often exhibits aberrant signaling pathways involving tyrosine kinases. Given that c-Abl is implicated in various cellular processes, including proliferation and survival, GNF2's ability to inhibit c-Abl could provide therapeutic benefits in ccRCC. The specific effects of GNF2 on ccRCC cells have not been thoroughly investigated, but its mechanism suggests potential utility in this context [51, 52]. Overall, our work provides new choices for better management of ccRCC.

Immunotherapy has provided new treatment options for advanced renal cancer and revolutionized the therapeutic paradigm for this type of tumor. The FDA has approved the combination of anti-PD1 or anti-CTLA4 antibodies with targeted therapy as a first-line treatment for advanced RCC [53]. However, it is important to note that most patients are not sensitive to ICI treatment, and severe immune-related adverse events can jeopardize patient health. Therefore, it is necessary to establish new classifications to identify specific renal cancer populations suitable for immunotherapy. Our study suggests that the XPS2 subgroup exhibits an immune exclusion phenotype, characterized by significantly lower infiltration of immune components such as CD8 T cells and macrophages compared to the XPS1 subtype, along with a lower response rate to immunotherapy. Identifying patient subgroups sensitive to ICIs is a current research focus in renal cancer. Our analysis based on the XPS classification revealed that the XPS1 subtype had a higher response rate to ICIs, while the XPS2 subtype had higher TIDE and immune dysfunction scores, correlating with a lower response rate to ICIs. Moreover, most immune-related regulatory factors, including chemokines, MHC, immune inhibitors, and stimulators, were relatively downregulated in XPS2, with only a few factors, such as TNFSF14 and TMIGD2, being upregulated. Meanwhile, previous works have mainly focused on the role of exportin family members in cancer cells, while the role of exportins in immune and stromal components has not been well investigated. Recently, Daneshmandi et al. discovered that XPO1 was upregulated in MDSCs in tumors, and XPO1 inhibition could maintain the nuclear residency of ERK1/2, thus reducing the level of phosphorylation triggered by the IL6/MAPK signal. Similarly, XPO1 blockade in human MDSCs induces the formation of neutrophil-like cells with immunostimulatory functions [54]. Based on these findings, we speculate that abnormally high expression of exportin family proteins may inhibit the expression of immune regulatory factors, ultimately inducing an immune exclusion phenotype. In the future, targeting exportin family proteins could potentially enhance the efficacy of ICIs in advanced renal cancer.

Although the XPS classification system effectively distinguishes different ccRCC patient prognosis risks and treatment sensitivities, this study still has some limitations. Firstly, despite including our in-house cohort, all the data used in this study are from retrospective cohorts, and the robustness of the model still needs to be validated in larger prospective cohorts. Secondly, since multiomics data are only available in some cohorts, further investigations are needed to determine whether XPS1 and XPS2 subgroups maintain stable heterogeneity in other omic layers in other cohorts. Thirdly, whether the XPS classification is applicable to advanced renal cancer patients receiving adjuvant therapy needs to be validated in specific cohorts. Lastly, this study is based on bioinformatics analysis, and more in vitro and in vivo experiments are required to validate the research results.

## 5. Conclusion

In summary, our study integrates multiomics data to systematically delineate the role of the exportin gene family in tumor progression and related biological mechanisms across pan-cancer and multiple ccRCC cohorts. We defined new ccRCC molecular subtypes and validated the reliability of the classification in local and public cohorts. Specifically, patients classified as XPS2 have a worse prognosis and exhibit characteristics such as immune exclusion, ICI treatment resistance, and genomic instability. XPO1 is abnormally expressed in KIRC and can promote renal cancer progression through the cell cycle, interferon, and TGF-beta-related pathways. In conclusion, this study provides new insights for the precise management of ccRCC patients from the perspective of the exportin gene family.

## Data Availability

The data that support the findings of this study are summarized in the Methods and Materials section.

## Nomenclature

ccRCC: clear cell renal cell carcinoma
SNVs: single nucleotide variations
CNVs: copy number variations
RCC: renal cell carcinoma
TKIs: tyrosine kinase inhibitors
mTOR: mammalian target of rapamycin
CRM1: Chromosomal Maintenance 1
CNAs: copy number alterations
CDF: cumulative distribution function
NTP: nearest template prediction
ssGSEA: single-sample gene set enrichment analysis
TIDE: tumor immune dysfunction and exclusion
FGA: percentage of genome alteration
FGG: fraction of genomic gain
FGL: fraction of genome loss
GDSC: Genomics of Drug Sensitivity in Cancer
CMAP: Connectivity Map
IC50: half-maximal inhibitory concentration
FDR: false discovery rate
TCGA: The Cancer Genome Atlas
GTEx: Genotype-Tissue Expression
KIRC: kidney renal clear cell carcinoma
KIRP: kidney renal papillary cell carcinoma
LUSC: lung squamous cell carcinoma
PAAD: pancreatic adenocarcinoma
THCA: thyroid carcinoma
GSVA: gene set variation analysis
KICH: kidney chromophobe
OS: overall survival
UVM: uveal melanoma
LIHC: liver hepatocellular carcinoma
BLCA: bladder urothelial carcinoma
XPS1: XPO-based subtype 1
MSI: microsatellite instability
MDSC: myeloid-derived suppressor cell
TIME: tumor immunity in the microenvironment
Th2: T helper 2 cell
ICI: immune checkpoint inhibitor
CCLE: Cancer Cell Line Encyclopedia
Treg: regulatory T cell

## Supporting Information

Supporting Information 1 Figure S1: Impact of exportin family genes on the prognosis of pan-cancer patients, including (A) progression-free interval, (B) disease-specific survival, and (C) disease-free interval.

Supporting Information 2 Figure S2: Representation of the XPS classification and Kaplan–Meier plot in (A, B) GSE22541, (C, D) CH-RCC, and (E, F) RECA-EU cohorts.

Supporting Information 3 Figure S3: Heatmaps showing (A) metabolic and (B) immune-related signaling differences between XPS1 and XPS2 subtypes.

Supporting Information 4 Figure S4: Heatmaps illustrating the differences in (A) immune cell infiltration and (B) immune-related features between XPS1 and XPS2 subtypes.

Supporting Information 5 Figure S5: Prediction of different target-based treatment IC50 values between XPS1 and XPS2 subtypes.

Supporting Information 6 Figure S6: Functional and communication differences between XPO high and XPO low macrophages. (A) Heatmap showing GSVA analysis results highlighting pathway differences between XPO high macrophages and XPO low macrophages. (B) Chord diagram illustrating cell–cell communication patterns derived from CellChat analysis. (C) Chord diagram showing the expression patterns of intercellular communication molecules based on CellCall analysis.

Supporting Information 7 Table S1: Summary of TCGA and GTEx sample sizes of different tumor types in this study. Abbreviations: ACC, adrenocortical cancer; BLCA, bladder urothelial carcinoma; BRCA, breast cancer; CESC, cervical cancer; CHOL, bile duct cancer; COAD, colon adenocarcinoma; DLBC, large B cell lymphoma; ESCA, esophageal cancer; GBM, glioblastoma; HNSC, head and neck squamous cell carcinoma; KICH, kidney chromophobe; KIRC, kidney renal clear cell carcinoma; KIRP, kidney renal papillary cell carcinoma; LAML, acute myeloid leukemia; LGG, lower grade glioma; LIHC, liver hepatocellular carcinoma; LUAD, lung adenocarcinoma; LUSC, lung squamous cell carcinoma; MESO, mesothelioma; OV, ovarian cancer; PAAD, pancreatic adenocarcinoma; PCPG, pheochromocytoma and paraganglioma; PRAD, prostate adenocarcinoma; READ, rectum adenocarcinoma; SARC, sarcoma; SKCM, skin cutaneous melanoma; STAD, stomach adenocarcinoma; TGCT, testicular cancer; THCA, thyroid carcinoma; THYM, thymoma; UCEC, uterine corpus endometrial carcinoma; UCS, uterine carcinosarcoma; UVMs, uveal melanomas.

Supporting Information 8 Table S2: Baseline information of ccRCC cohort.

Supporting Information 9 Table S3: Difference in clinical information between XPS1 and XPS2.
