# Supplementary figures and images for: Multiomics Analysis of Exportin Family Reveals XPO1 as a Novel Target for Clear Cell Renal Cell Carcinoma
Source: Int J Genomics. 2025 Jan 21;2025:3645641. doi: 10.1155/ijog/3645641 (PMC11774578; doi:10.1155/ijog/3645641)

## Progression Free Interval

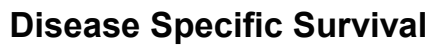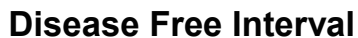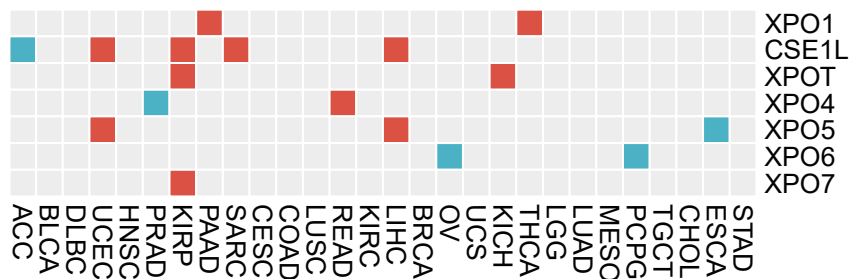

Supplement: Supporting Information 1 — Figure S1: Impact of exportin family genes on the prognosis of pan-cancer patients, including (A) progression-free interval, (B) disease-specific survival, and (C) disease-free interval. [file 3645641.f1.pdf]

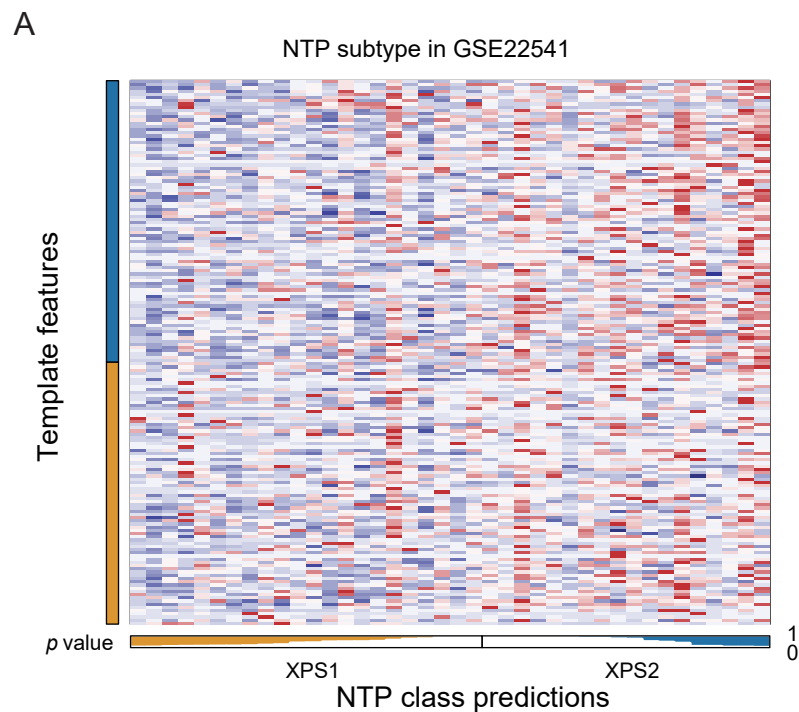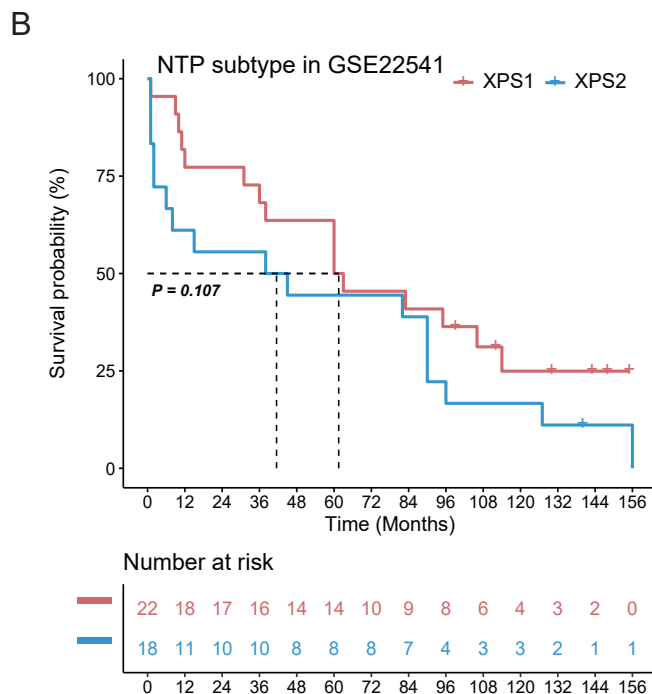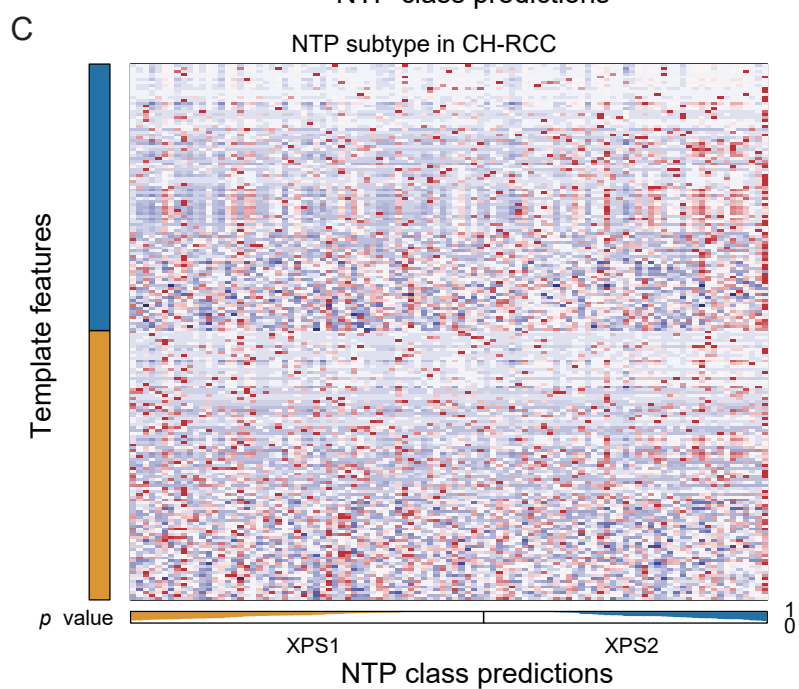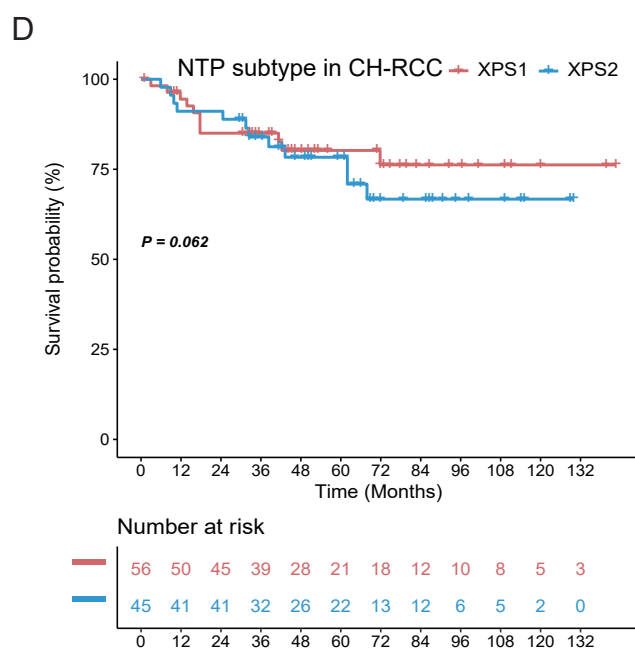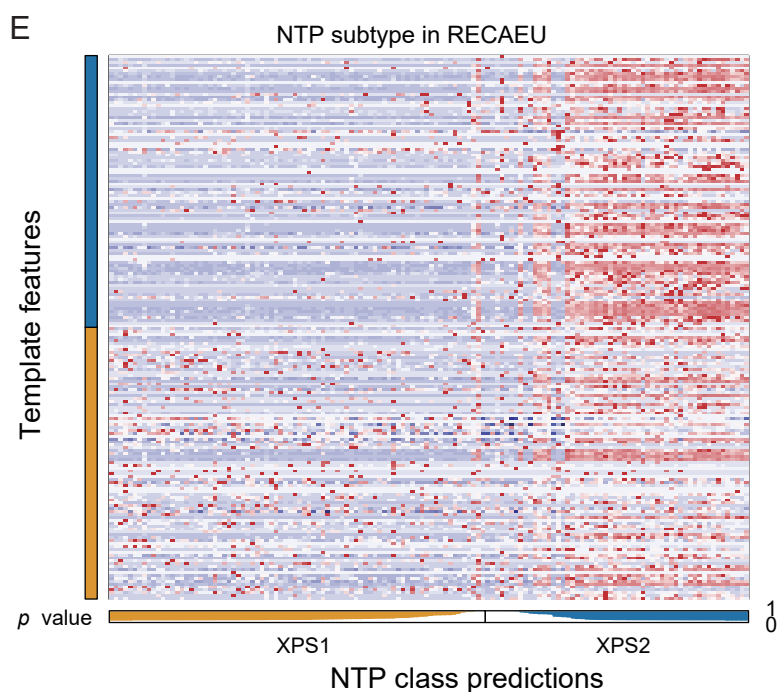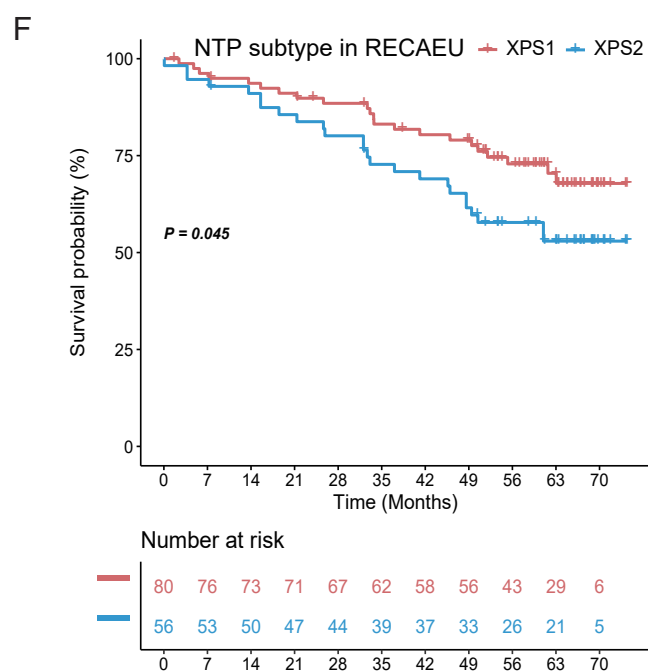

Supplement: Supporting Information 2 — Figure S2: Representation of the XPS classification and Kaplan–Meier plot in (A, B) GSE22541, (C, D) CH-RCC, and (E, F) RECA-EU cohorts. [file 3645641.f2.pdf]

A

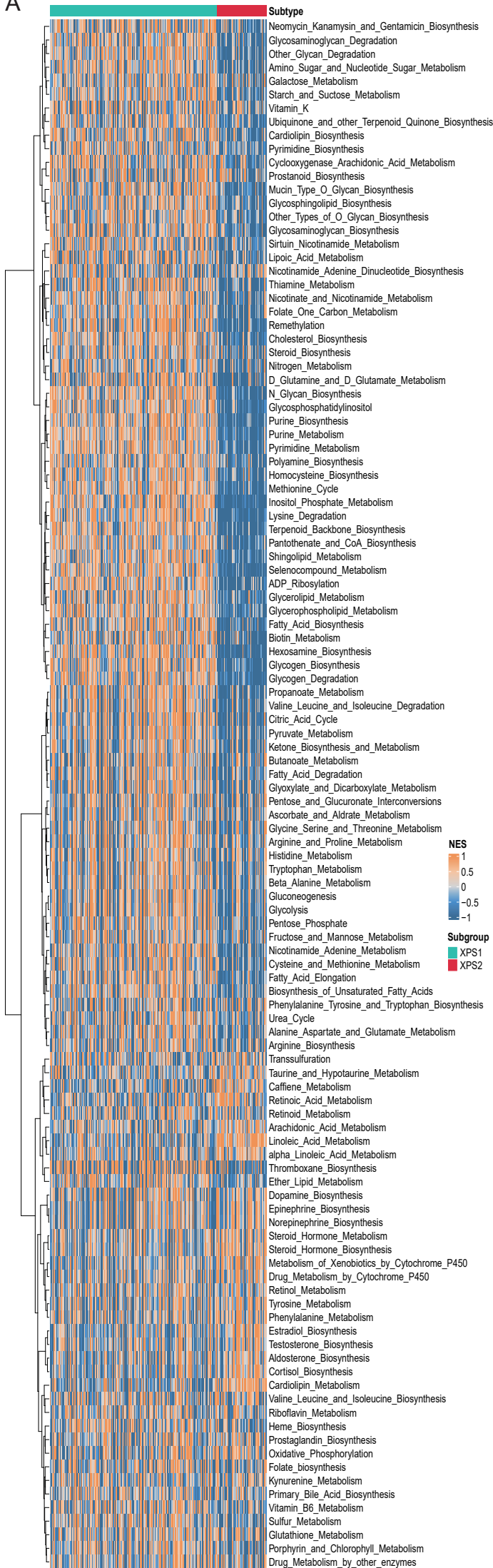

B

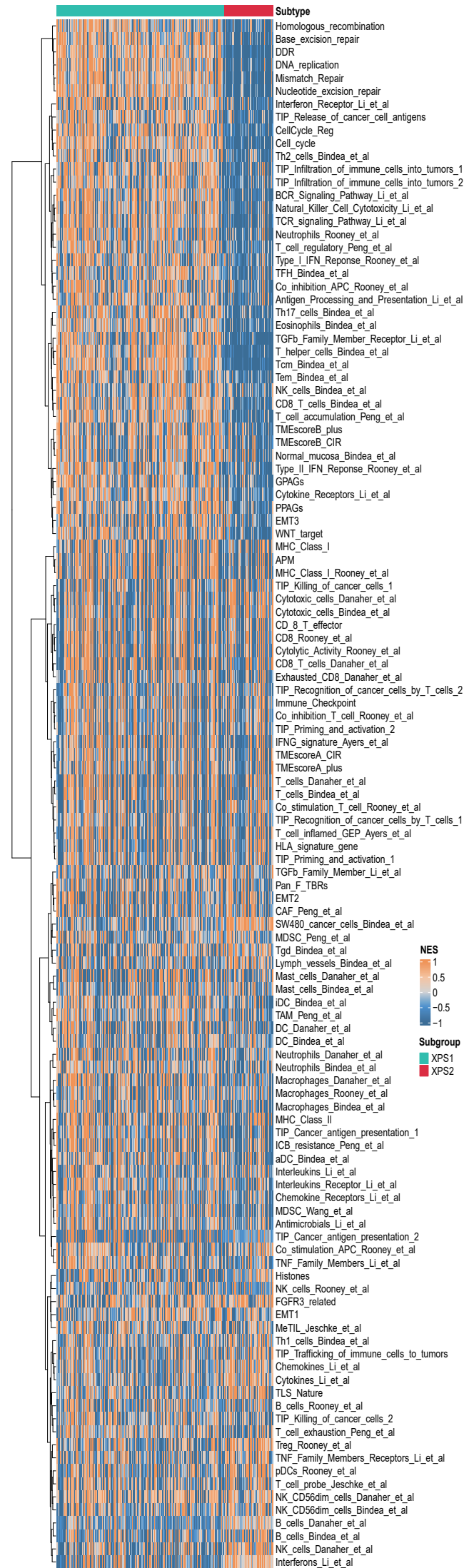

Supplement: Supporting Information 3 — Figure S3: Heatmaps showing (A) metabolic and (B) immune-related signaling differences between XPS1 and XPS2 subtypes. [file 3645641.f3.pdf]

A

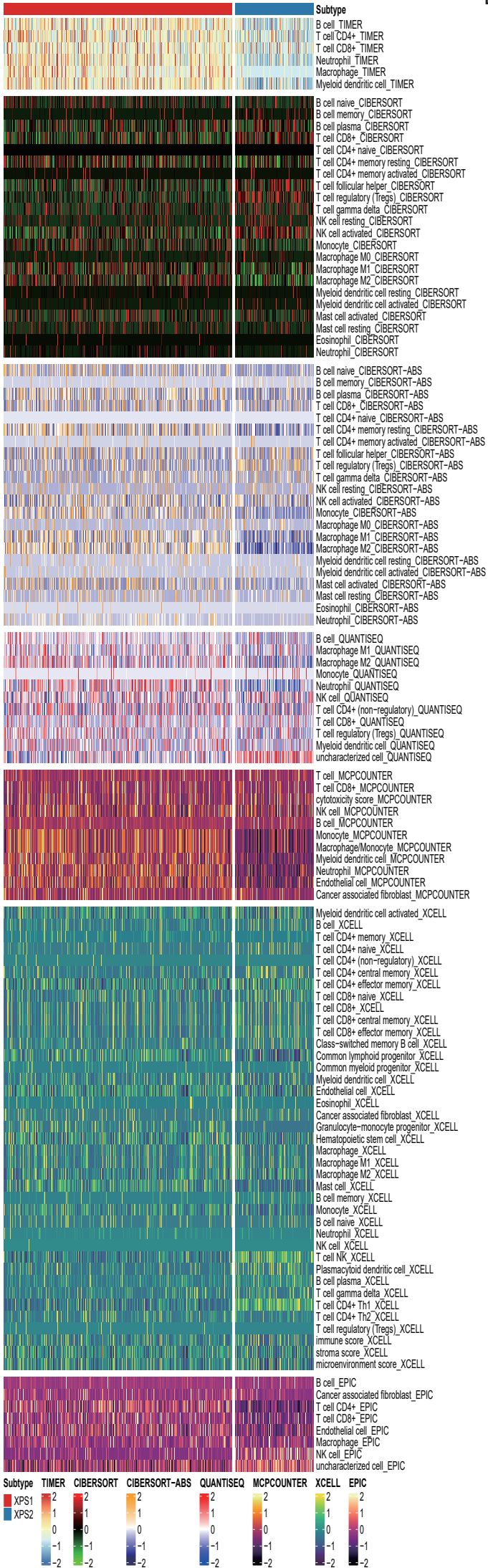

B

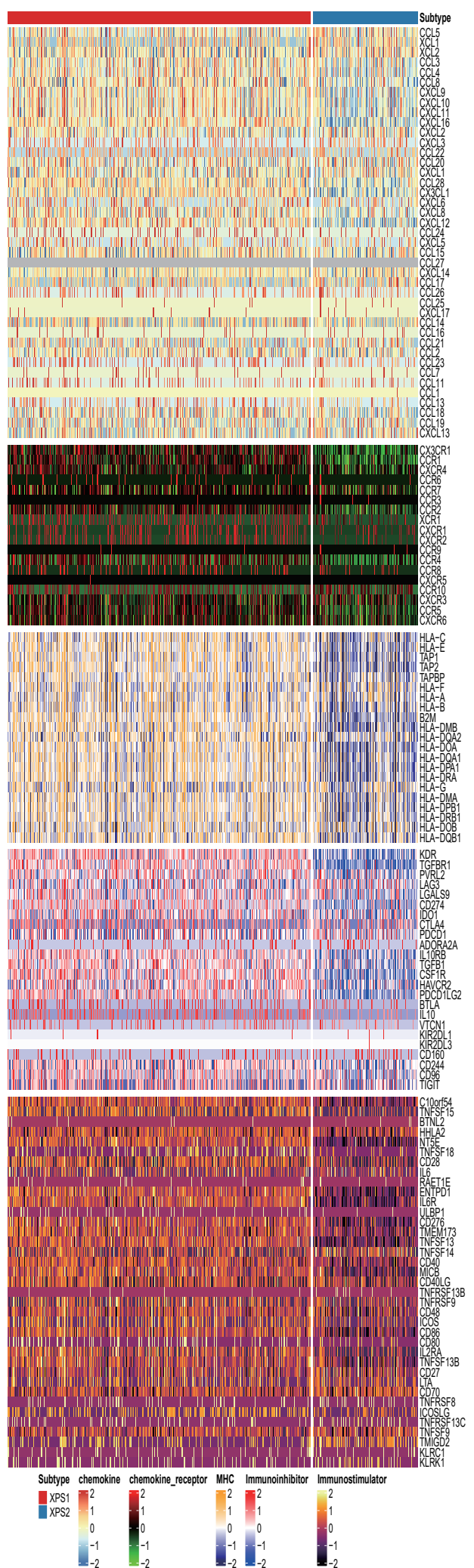

Supplement: Supporting Information 4 — Figure S4: Heatmaps illustrating the differences in (A) immune cell infiltration and (B) immune-related features between XPS1 and XPS2 subtypes. [file 3645641.f4.pdf]

A

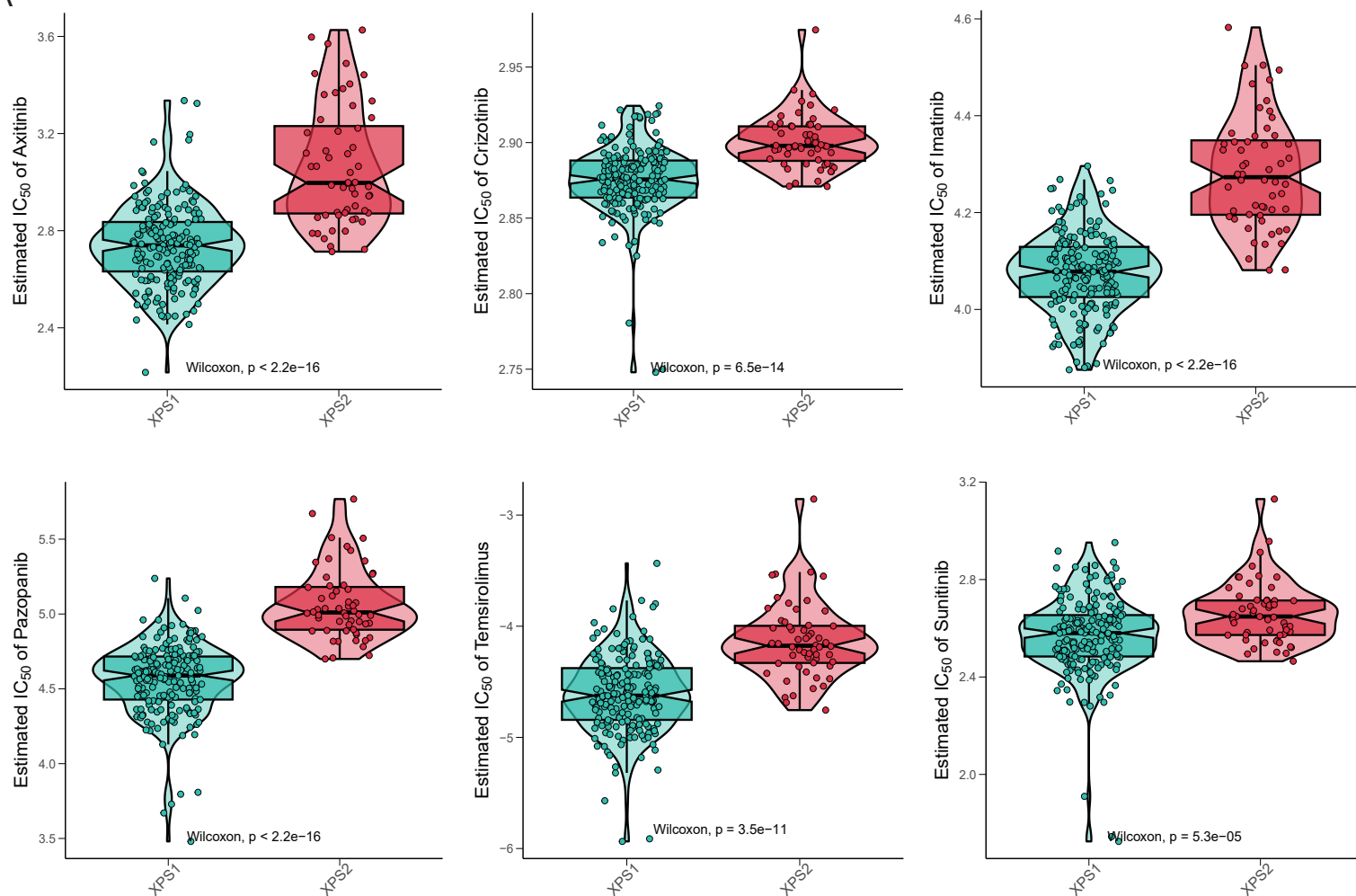

B

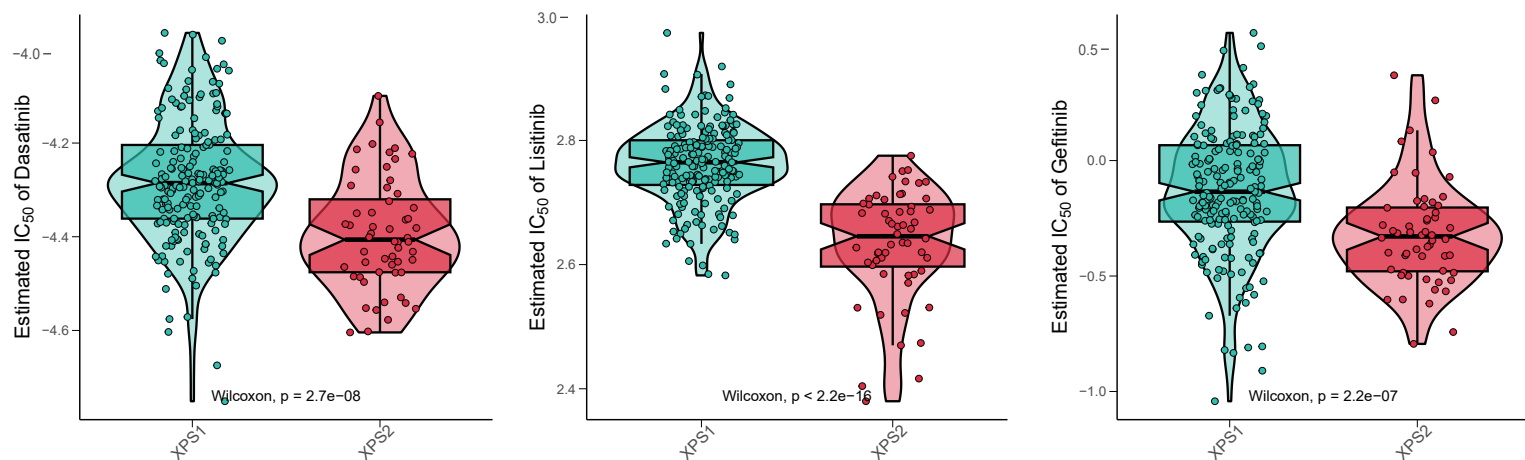

Supplement: Supporting Information 5 — Figure S5: Prediction of different target-based treatment IC50 values between XPS1 and XPS2 subtypes. [file 3645641.f5.pdf]

A

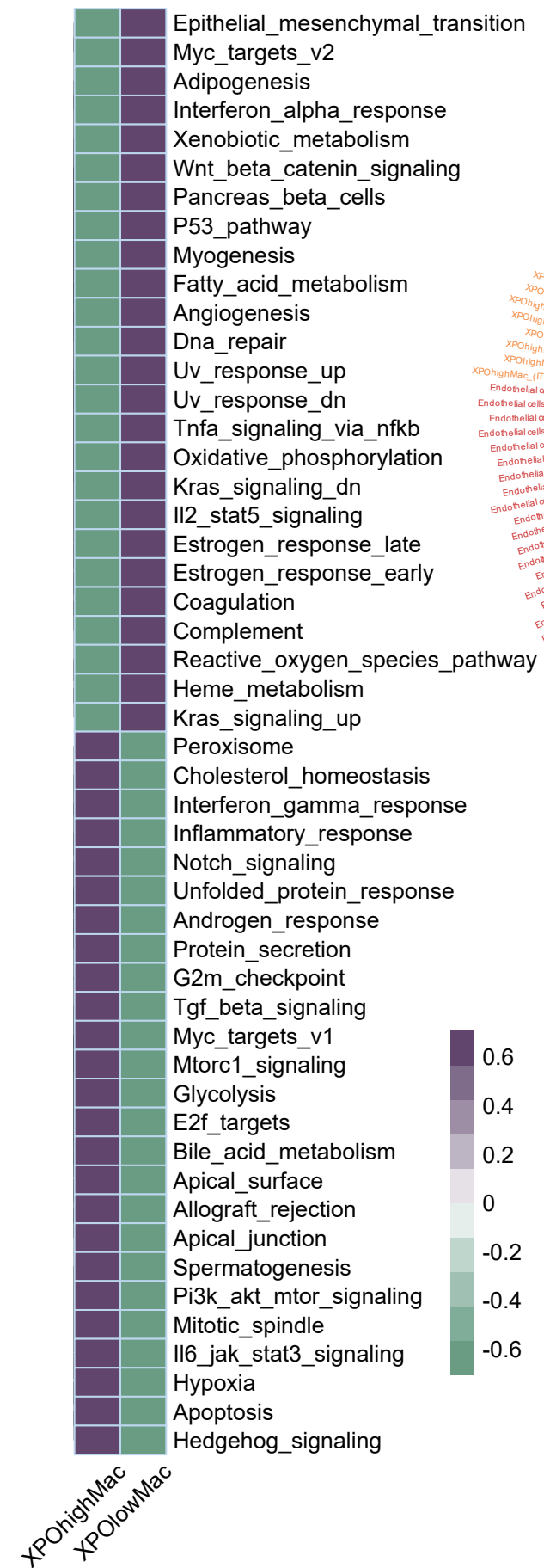

B

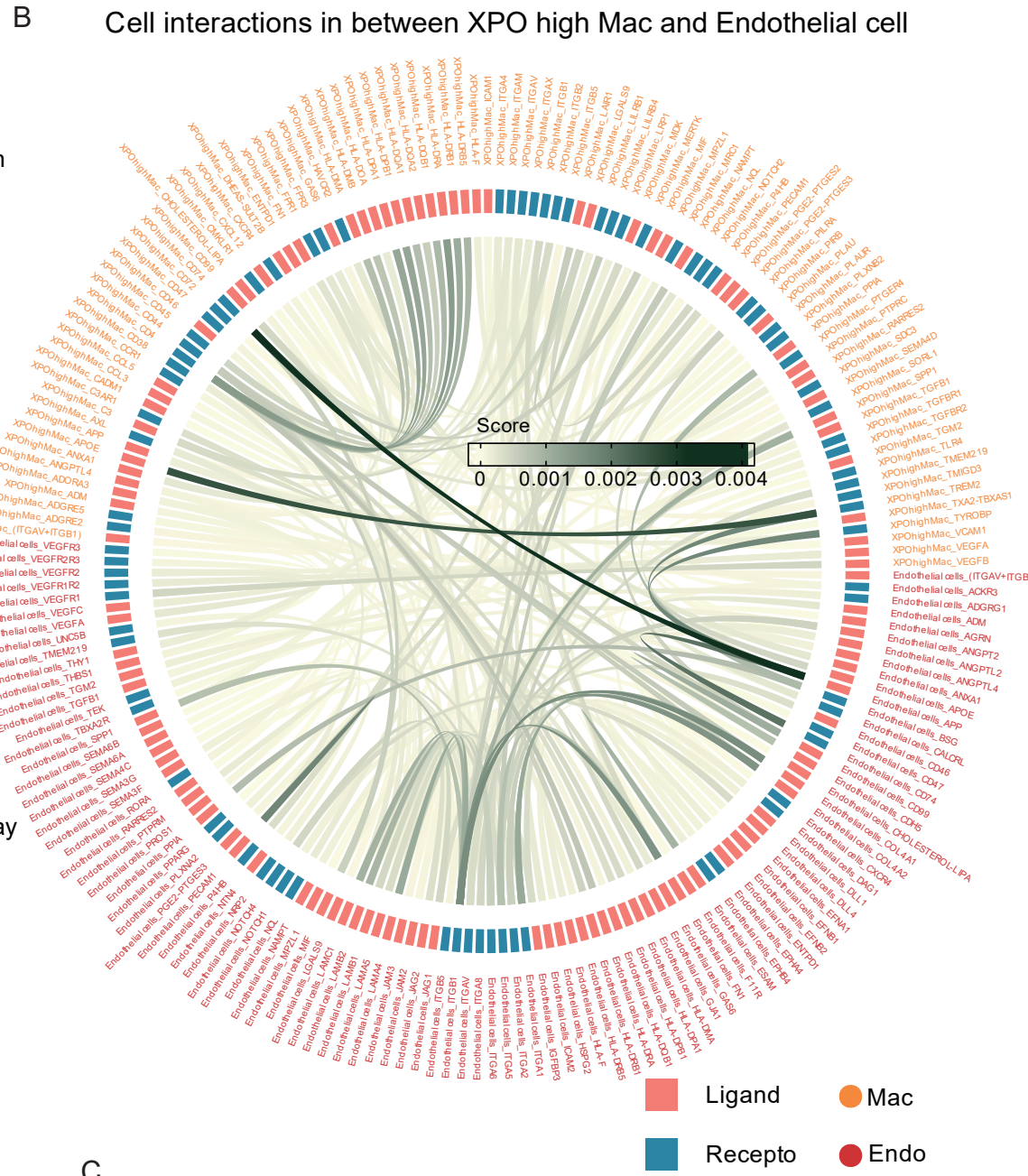

C

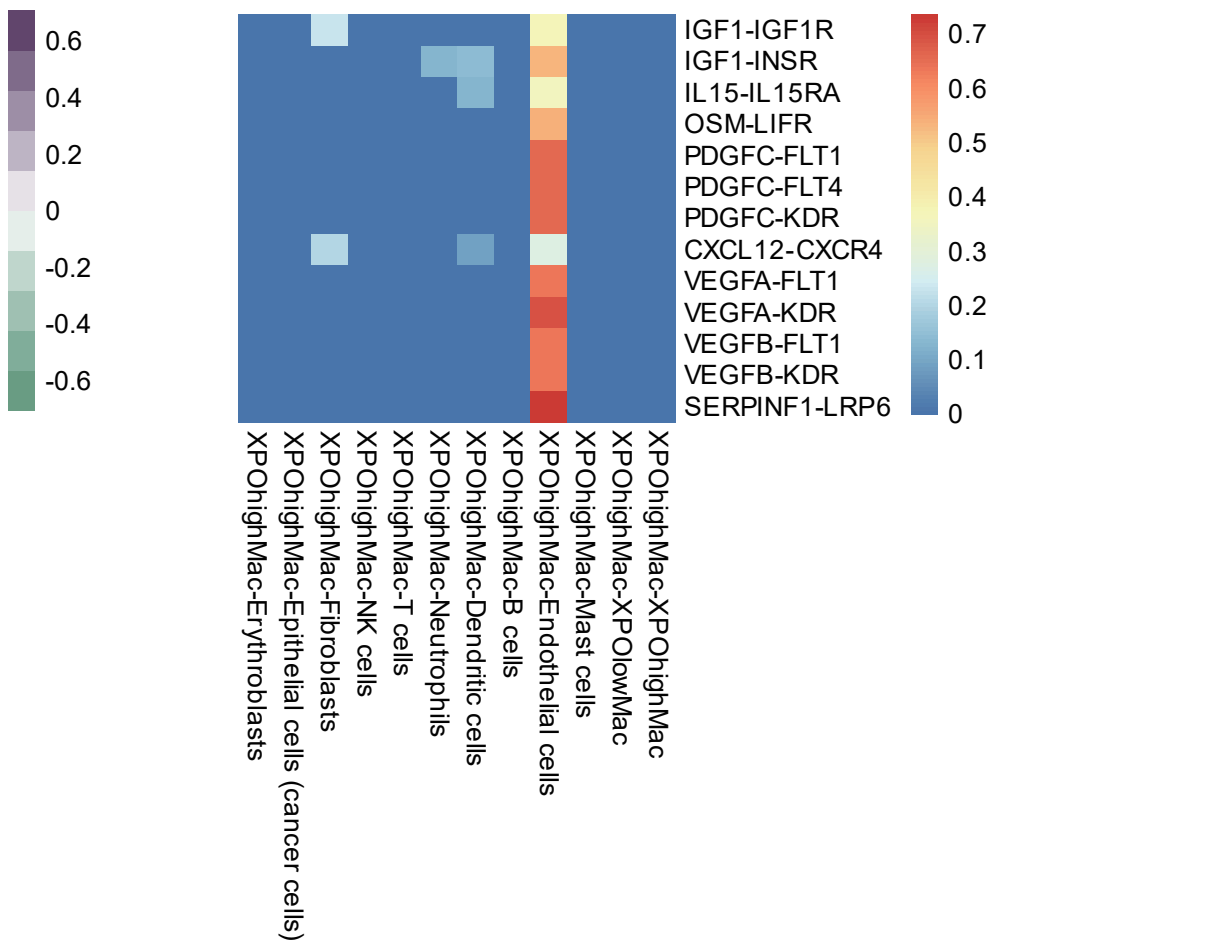

Supplement: Supporting Information 6 — Figure S6: Functional and communication differences between XPO high and XPO low macrophages. (A) Heatmap showing GSVA analysis results highlighting pathway differences between XPO high macrophages and XPO low macrophages. (B) Chord diagram illustrating cell–cell communication patterns derived from CellChat analysis. (C) Chord diagram showing the expression patterns of intercellular communication molecules based on CellCall analysis. [file 3645641.f6.pdf]
