# Supplementary material for: Multiomics Analysis of Exportin Family Reveals XPO1 as a Novel Target for Clear Cell Renal Cell Carcinoma
Source: Int J Genomics. 2025 Jan 21;2025:3645641. doi: 10.1155/ijog/3645641 (PMC11774578; doi:10.1155/ijog/3645641)
Supplement: Supporting Information 7 — Table S1: Summary of TCGA and GTEx sample sizes of different tumor types in this study. Abbreviations: ACC, adrenocortical cancer; BLCA, bladder urothelial carcinoma; BRCA, breast cancer; CESC, cervical cancer; CHOL, bile duct cancer; COAD, colon adenocarcinoma; DLBC, large B cell lymphoma; ESCA, esophageal cancer; GBM, glioblastoma; HNSC, head and neck squamous cell carcinoma; KICH, kidney chromophobe; KIRC, kidney renal clear cell carcinoma; KIRP, kidney renal papillary cell carcinoma; LAML, acute myeloid leukemia; LGG, lower grade glioma; LIHC, liver hepatocellular carcinoma; LUAD, lung adenocarcinoma; LUSC, lung squamous cell carcinoma; MESO, mesothelioma; OV, ovarian cancer; PAAD, pancreatic adenocarcinoma; PCPG, pheochromocytoma and paraganglioma; PRAD, prostate adenocarcinoma; READ, rectum adenocarcinoma; SARC, sarcoma; SKCM, skin cutaneous melanoma; STAD, stomach adenocarcinoma; TGCT, testicular cancer; THCA, thyroid carcinoma; THYM, thymoma; UCEC, uterine corpus endometrial carcinoma; UCS, uterine carcinosarcoma; UVMs, uveal melanomas. [file 3645641.f7.docx]

**Table S1. Summary of TCGA and GTEx sample sizes of different tumor types in this study.** Abbreviations: ACC, Adrenocortical Cancer; BLCA, Bladder Cancer; BRCA, Breast Cancer; CESC, Cervical Cancer; CHOL, Bile Duct Cancer; COAD, Colon Adenocarcinoma; DLBC, Large B-cell Lymphoma; ESCA, Esophageal Cancer; GBM, Glioblastoma; HNSC, Head and Neck Squamous Cell Carcinoma; KICH, Kidney Chromophobe; KIRC, Kidney Renal Clear Cell Carcinoma; KIRP, Kidney Renal Papillary Cell Carcinoma; LAML, Acute Myeloid Leukemia; LGG, Lower Grade Glioma; LIHC, Liver Hepatocellular Carcinoma; LUAD, Lung Adenocarcinoma; LUSC, Lung Squamous Cell Carcinoma; MESO, Mesothelioma; OV, Ovarian Cancer; PAAD, Pancreatic Cancer; PCPG, Pheochromocytoma & Paraganglioma; PRAD, Prostate Adenocarcinoma; READ, Rectum Adenocarcinoma; SARC, Sarcoma; SKCM, Skin Cutaneous Melanoma; STAD, Stomach Adenocarcinoma; TGCT, Testicular Cancer; THCA, Thyroid Cancer; THYM, Thymoma; UCEC, Uterine Corpus Endometrial Carcinoma; UCS, Uterine Carcinosarcoma; UVM, Ocular melanomas.

| Tumor type | GTEx (Normal) | TCGA (Normal) | TCGA (Tumor) |
| --- | --- | --- | --- |
| ACC | 258 | 0 | 89 |
| BLCA | 21 | 19 | 406 |
| BRCA | 459 | 113 | 1101 |
| CESC | 19 | 3 | 306 |
| CHOL | 0 | 9 | 35 |
| COAD | 779 | 41 | 455 |
| DLBC | 929 | 0 | 48 |
| ESCA | 1445 | 11 | 163 |
| GBM | 2642 | 5 | 153 |
| HNSC | 0 | 44 | 504 |
| KICH | 89 | 25 | 65 |
| KIRC | 89 | 72 | 532 |
| KIRP | 89 | 32 | 290 |
| LAML | 0 | 0 | 150 |
| LGG | 2642 | 0 | 513 |
| LIHC | 226 | 50 | 371 |
| LUAD | 578 | 59 | 516 |
| LUSC | 578 | 49 | 501 |
| MESO | 0 | 0 | 89 |
| OV | 180 | 0 | 376 |
| PAAD | 328 | 4 | 179 |
| PCPG | 0 | 3 | 181 |
| PRAD | 245 | 52 | 498 |
| READ | 779 | 10 | 165 |
| SARC | 0 | 2 | 260 |
| SKCM | 1809 | 1 | 471 |
| STAD | 359 | 32 | 375 |
| TGCT | 361 | 0 | 134 |
| THCA | 653 | 59 | 512 |
| THYM | 0 | 2 | 120 |
| UCEC | 142 | 35 | 545 |
| UCS | 142 | 0 | 57 |
| UVM | 0 | 0 | 80 |
