# Supplementary material for: Multiomics Analysis of Exportin Family Reveals XPO1 as a Novel Target for Clear Cell Renal Cell Carcinoma
Source: Int J Genomics. 2025 Jan 21;2025:3645641. doi: 10.1155/ijog/3645641 (PMC11774578; doi:10.1155/ijog/3645641)
Supplement: Supporting Information 8 — Table S2: Baseline information of ccRCC cohort. [file 3645641.f8.docx]

| **Table S2 Base lines information of ccRCC cohort** | | | |  |  |
| --- | --- | --- | --- | --- | --- |
| **Different cohorts** | **TCGA−KIRC** | **ICGC-EU** | **GSE22541** | | **Changhai Cohort** |
| **Number of samples** | 530 | 91 | 40 | | 84 |
| **Gender** |  |  |  |  |  |
| male | 344 | 39 | 24 | | 32 |
| female | 186 | 52 | 16 | | 52 |
| **Age, years** |  |  |  | |  |
| Mean ± SD | 60.6 ± 12.1 | 60.5 ± 10.0 | − | |  |
| **T** |  |  |  |  |  |
| T1 | 271 | 54 | − | | 25 |
| T2 | 69 | 13 | − | | 26 |
| T3 | 179 | 22 | − | | 20 |
| T4 | 11 | 2 | − | | 13 |
| unknow | **−** | **−** |  | | 0 |
| **N** |  |  |  |  |  |
| N0 | 239 | 79 | − | | 54 |
| N1 | 16 | 2 | − | | 30 |
| unknown | 275 |  | − | | 0 |
| **M** |  |  |  |  |  |
| M0 | 420 | 81 | **−** | | 75 |
| M1 | 78 | 9 | **−** | | 5 |
| unknown | 32 | 1 | **−** | | 4 |
| **Stage** |  |  |  |  |  |
| I | 14 | 9 | − | | 22 |
| II | 227 | 48 | − | | 23 |
| III | 208 | 12 | − | | 24 |
| IV | 75 | 13 | − | | 15 |
| unknow | 6 | 9 | − | | **−** |
| **Download Links** | [**https://pdc.cancer.gov/pdc/**](https://pdc.cancer.gov/pdc/) | [**https://dcc.icgc.org/**](https://dcc.icgc.org/) | [**https://www.ncbi.nlm.nih.gov/geo/query/acc.cgi?acc=GSE22541**](https://www.ncbi.nlm.nih.gov/geo/query/acc.cgi?acc=GSE22541) | | **−** |
