# Supplementary material for: Multiomics Analysis of Exportin Family Reveals XPO1 as a Novel Target for Clear Cell Renal Cell Carcinoma
Source: Int J Genomics. 2025 Jan 21;2025:3645641. doi: 10.1155/ijog/3645641 (PMC11774578; doi:10.1155/ijog/3645641)
Supplement: Supporting Information 9 — Table S3: Difference in clinical information between XPS1 and XPS2. [file 3645641.f9.docx]

Table S3 Difference in clinical information between XPS1 and XPS2

|  | XPS1 | XPS2 | p.overall |
| --- | --- | --- | --- |
|  | *N=380* | *N=131* |  |
| T: |  |  | 0.358 |
| T1 | 204 (53.7%) | 60 (45.8%) |  |
| T2 | 47 (12.4%) | 21 (16.0%) |  |
| T3 | 120 (31.6%) | 48 (36.6%) |  |
| T4 | 9 (2.37%) | 2 (1.53%) |  |
| N: |  |  | 0.770 |
| N1 | 10 (4.95%) | 5 (6.17%) |  |
| NX | 192 (95.0%) | 76 (93.8%) |  |
| stage: |  |  | 0.336 |
| i | 200 (52.6%) | 58 (44.3%) |  |
| ii | 42 (11.1%) | 14 (10.7%) |  |
| iii | 81 (21.3%) | 36 (27.5%) |  |
| iv | 57 (15.0%) | 23 (17.6%) |  |
| sex: |  |  | 0.440 |
| female | 135 (35.5%) | 41 (31.3%) |  |
| male | 245 (64.5%) | 90 (68.7%) |  |
| age | 60.3 (12.2) | 60.8 (12.3) | 0.642 |
| OS: |  |  | 0.005 |
| 0 | 268 (70.5%) | 74 (56.5%) |  |
| 1 | 112 (29.5%) | 57 (43.5%) |  |
| OS.time | 1413 (989) | 1350 (926) | 0.511 |
| PFI: |  |  | 0.016 |
| 0 | 274 (72.1%) | 79 (60.3%) |  |
| 1 | 106 (27.9%) | 52 (39.7%) |  |
| PFI.time | 1218 (960) | 1158 (884) | 0.509 |
